# Modulation of Autophagy by Ursolic and Betulinic Acids: Distinct Cytotoxic and Membrane‐Disruption in Malignant and Nonmalignant Cells

**DOI:** 10.1002/cbin.70073

**Published:** 2025-08-23

**Authors:** Waleska Kerllen Martins, Tayana Mazin Tsubone, Chimara Emilia Nascimento Sanches, Cleidiane de Sousa Rocha, Ricardo Scarparo Navarro, Beatriz Simonsen Stolf, Susana Nogueira Diniz, Rosangela Itri, Mauricio S. Baptista

**Affiliations:** ^1^ Instituto de Química Universidade de São Paulo São Paulo São Paulo Brazil; ^2^ Bioengenharia, Instituto Científico e Tecnológico Universidade Brasil São Paulo São Paulo Brazil; ^3^ Instituto de Física Universidade de São Paulo São Paulo São Paulo Brazil; ^4^ Instituto de Química Universidade Federal de Uberlândia Uberlândia Minas Gerais Brazil; ^5^ Instituto de Ciências Biomédicas Universidade de São Paulo São Paulo São Paulo Brazil; ^6^ Centro Universitário Anhanguera de São Paulo São Paulo São Paulo Brazil

**Keywords:** autophagy modulation, autophagy‐associated cell death, mimetic‐membrane models, mitochondrial–lysosomal stress axis, triterpenoids

## Abstract

Autophagy is a critical adaptive mechanism in tumor cells that promotes survival under stress, but when dysregulated, it may trigger programmed cell death. The pentacyclic triterpenoids betulinic acid (BA) and ursolic acid (UA) are structurally related compounds that modulate autophagy; however, comparative insights into their effects on nonmalignant and malignant cells, as well as model membranes, remain limited. Here, we investigated the distinct cellular outcomes induced by UA and BA in nonmalignant keratinocytes (HaCaT) and malignant cell lines (A549, HeLa, MCF7, MES‐SA, PC3, SKMEL‐25/28), as well as their interactions with mitochondrial membrane mimetics. At 20 μM, BA reduced HaCaT proliferation by 70%, while UA achieved only 30% inhibition. BA induced pronounced mitochondrial dysfunction (i.e., 60%), mitophagy activation, and autophagy‐associated cell death linked to a lysosomal–mitochondrial stress axis. In contrast, UA induced lysosomal membrane permeabilization and the release of cathepsin B, resulting in ~50% cell death. In malignant cell lines, BA reduced viability to ~40%, whereas UA showed selective toxicity (53%–73% survival). Cotreatment with chloroquine enhanced UA's cytotoxicity by simulating BA‐like lysosomal accumulation. Biophysical assays revealed differential membrane disruption profiles: BA permeabilized cardiolipin‐rich membranes, while UA exerted milder surface‐level effects. These findings illustrate how structurally similar triterpenoids exert divergent effects on cellular membranes, autophagic flux, and cell fate, offering a foundation for designing selective anticancer agents that target the lysosomal–mitochondrial axis.

**Abbreviations**
AACD: autophagy‐associated cell death
AKT: AKT serine/threonine kinase 1
AMPK: AMP‐activated protein kinase
ASM: acid sphingomyelinase
ATG: autophagy‐related
AVOs: acidic vesicle organelles
BA: betulinic acid
BMP: bis(monoacylglycero)phosphate
BP: bandpass
CCCP: carbonyl cyanide m‐chlorophenylhydrazone
COXIV: cytochrome c oxidase (COX) subunit IV
CQ: chloroquine
CTSB: cathepsin B
CTSL: cathepsin L
CVS: crystal violet staining
DAPI: 4′,6‐diamidino‐2‐phenylindole
GAPDH: glyceraldehyde‐3‐phosphate dehydrogenase
GUV: giant unilamellar vesicle
HaCaT: human immortalized keratinocyte cell line
LC3B‐II: microtubule‐associated protein 1 light chain 3 beta‐II
LMP: lysosomal membrane permeabilization
LTR: LysoTracker Red DND‐99
MTG: MitoTracker Green FM
mTOR: mechanistic target of rapamycin kinase
MTR: MitoTracker Red CM‐H_2_XRos
MTT: methylthiazolyldiphenyl‐tetrazolium bromide
OA: oleanolic acid
PBS: phosphate‐buffered saline
PI: propidium iodide
PI3K: phosphoinositide‐3‐kinase
PINK1: PTEN‐induced putative kinase 1
PRKN: parkin RBR E3 ubiquitin‐protein ligase
Rh123: rhodamine 123
ROS: reactive oxygen species
UA: ursolic acid
ΔΨm: mitochondrial membrane potential

## 1 Introduction

Cancer is a significant burden characterized by a complex and multifactorial nature, which may culminate in aggressive, therapeutically multiresistant behavior (Hanahan 2022). Its incidence is rising in both developed and emerging nations, reaching over 35 million cases by 2050, mainly due to population aging and increased exposure to risk factors (Ferlay et al. 2024). These alarming trends underscore the urgent need for effective antitumor strategies to improve clinical outcomes.

Macroautophagy, hereafter referred to as autophagy, has emerged as a promising therapeutic target, especially for modulating chemoresistance or chemosensitivity (Martins et al. 2021). As a primary catabolic process responsible for the degradation of macromolecules and organelles, autophagy can promote either cell survival or cell death, depending on the context (Klionsky et al. 2021; Mizushima and Levine 2020). Its regulation in cancer involves multiple pathways, including AMP‐activated protein kinase (AMPK), mitogen‐activated protein kinase (MAPK), phosphoinositide‐3‐kinase (PI3K)/AKT serine–threonine kinase 1 (AKT), Beclin 1, ATG proteins, and noncoding RNAs (Qin et al. 2023).

Both canonical and noncanonical forms of autophagy ultimately rely on lysosomes for the degradation of cargo. This includes the selective clearance of defective mitochondria via mitophagy, a critical process for maintaining redox balance and mitochondrial quality control (Garza‐Lombó et al. 2020; Mizushima and Levine 2020). Upon mitochondrial depolarization, proteins such as PTEN‐induced putative kinase 1 (PINK1) and parkin RBR E3 ubiquitin‐protein ligase (PRKN) coordinate the ubiquitination of outer membrane components, recruiting adaptor proteins like SQSTM1/p62 and LC3‐II to facilitate autophagosome formation and cargo sequestration (Garza‐Lombó et al. 2020).

Autophagy enables tumor cells to resist various stresses, including nutrient deprivation, hypoxia, and damage induced by chemo‐, radio‐, and photodynamic therapy (Martins, Fader, et al. 2021; Martins, Silva, et al. 2021; Martins, Belotto, et al. 2021). However, when the autophagy process is persistently impaired, it can activate a lysosomal–mitochondrial stress axis leading to cell fate transitions such as autophagy‐associated cell death (AACD), senescence, or accumulation of lipofuscin (Martins et al. 2017, 2019; Tonolli et al. 2020; Tsubone et al. 2020). These prolonged effects, which can be modified by drugs such as natural compounds, underscore the potential of autophagy as a promising therapeutic target in various cancer cells (Martins, Silva, et al. 2021; Martins, Belotto, et al. 2021).

Among such agents, pentacyclic triterpenoids stand out for their multifaceted bioactivities. Ursolic acid (UA) and betulinic acid (BA) are two naturally occurring isomers found in various plants, including *Betula* species (BA) and fruits like cranberries and olives (UA) (National Center for Biotechnology Information 2024a, 2024b). Both have demonstrated anticancer effects and are known to modulate autophagy; however, their mechanisms differ across various cell types (El‐Baba et al. 2021). UA promotes protective autophagy in malignant cells via the PI3K/AKT/mTOR or MAPK signaling pathways, which may limit its cytotoxic effects unless used in combination with autophagy inhibitors (Castrejón‐Jiménez et al. 2019; Lin et al. 2019; Shin et al. 2012; M. Wang et al. 2020; Zhao et al. 2013). However, UA can modulate autophagy as a mechanism of cell death rather than a protective response, depending on the concentration and specific cellular context (Fogde et al. 2022; Lee et al. 2020; Leng et al. 2013; Lewinska et al. 2017; Shen et al. 2014; Z. Wang et al. 2022; Xavier et al. 2013).

BA often induces apoptotic cell death by suppressing PI3K/AKT/mTOR, or through autophagic flux blockage, causing accumulation of LC3‐II, SQSTM1/p62, and misfolded proteins pathway (F. Chen et al. 2020; Liu et al. 2019; Sun et al. 2021; Yang et al. 2012; S. Wang et al. 2017). Mitochondrial damage frequently serves as a key trigger for BA‐induced apoptosis, while acts as a temporary compensatory response (Potze et al. 2014; Sun et al. 2021; S. Wang et al. 2017). Additionally, BA can display a dual role—both inducing and impairing autophagic flux—likely through direct membrane interactions with the mitochondria and lysosomes of nonmalignant keratinocytes (Martins et al. 2015, 2017). Although UA has been studied in malignant contexts, little is known about its behavior in nonmalignant human cells, particularly keratinocytes—a critical gap given the relevance of autophagy in skin homeostasis and treatment toxicity.

This study aims to compare the autophagy‐related effects of UA and BA across nonmalignant keratinocytes (HaCaT) and seven human cancer cell lines, including A549, HeLa, MES‐SA, PC3, MCF7, SKMEL‐25, and SKMEL‐28. We explore how each compound modulates mitochondrial and lysosomal function, triggers distinct cell death pathways, and interacts with artificial membranes that mimic cellular bilayers. The findings offer mechanistic insight and translational value, contributing to the rational development of autophagy‐modulating anticancer strategies.

## 2 Material and Methods

### 2.1 Materials

BA (B8939) and UA (U6753) were purchased from Sigma‐Aldrich, and stock solutions of 4 mg/mL were prepared in 100% dimethyl sulfoxide (DMSO; Sigma‐Aldrich, D2650). Rhodamine 123 (Rh123, Sigma‐Aldrich, R8004), propidium iodide (PI; Sigma‐Aldrich, 81845), and acridine orange (AO; Sigma‐Aldrich, A6014) were dissolved in deionized water to create stock solutions of 2 mM or 1 mg/mL. The antibodies used were anti‐rabbit PRKN (Abcam, ab159594), anti‐rabbit LC3B (D11) XP (Cell Signaling Technology, 3868), anti‐mouse CTSB (Abcam, 58802), anti‐mouse COXIV (Invitrogen, A21347), anti‐mouse GAPDH (Sigma‐Aldrich, G9545), anti‐rabbit IgG (H + L) (Invitrogen, A‐11034 or A‐21070), and anti‐mouse IgG (Invitrogen, A‐11001 or A‐21050). LysoTracker Red DND‐99 (LTR, Invitrogen, L‐7528), MitoTracker Green FM (MTG, Invitrogen, M‐7514), and MitoTracker Red CM‐H_2_XRos (MTR, Invitrogen, M‐7513) were dissolved in DMSO to create 1 mM stock solutions. We used ProLong Gold antifade mounting with 4′,6‐diamidino‐2‐phenylindole (DAPI, Invitrogen, P36935). Piperazine‐*N*,*N*′‐bis(2‐ethane sulfonic acid) (PIPES, P1851), NaCl (S9888), ethylenediaminetetraacetic acid (EDTA, E9884), sucrose (S0389), 3‐[(3‐cholamidopropyl)dimethylammonio]‐1‐propanesulfonate hydrate (CHAPS, C3023), Triton X‐100 (T8787), polyethylene glycol sorbitan monolaurate (Tween 20, 274348), phenylmethylsulfonyl fluoride (PMSF, 78830), pepstatin A (77170), 5(6)‐carboxyfluorescein (CF, C0537), carbonyl cyanide *m*‐chlorophenylhydrazone (CCCP, C2759), chloroquine (CQ, 50635), crystal violet (CV, C6158), and digitonin (D141) were purchased from Sigma‐Aldrich.

### 2.2 Cell Lines and Cell Culture

Instituto Butantan (São Paulo, Brazil) gently supplied human immortalized keratinocyte cell line HaCaT, and Ludwig Institute for Cancer Research (São Paulo, Brazil) kindly provided the human carcinoma cells A549, HeLa, MES‐SA, PC3, and cutaneous melanoma (SKMEL‐25 and SKMEL‐28). These cells were cultured in Dulbecco Modified Eagle Medium (DMEM, Gibco, 12100046) with 10% (v/v) fetal bovine serum (Gibco, 12657029), 100 units/mL of penicillin, 100 μg/mL of streptomycin, and 250 ng/mL of amphotericin B in a 37°C incubator under a moist atmosphere of 5% carbon dioxide. We cultured MCF7 cells in DMEM /F‐12 without phenol red (Gibco, 21041025).

### 2.3 Cytotoxicity

To assess the survival rate over time, rather than examining immediate cytotoxic effects, human cell lines were maintained for two doubling times after treatment with BA or UA at concentrations ranging from 10 to 40 µM for 24 h. DMSO levels ranged from 0.125% to 0.50% (v/v). We performed crystal violet staining (CVS) and MTT assays independently (Martins et al. 2013).

### 2.4 Quantification of Lysosomal Accumulation

The method's conceptual framework was based on the higher uptake of neutral red (NRU) in cells undergoing death, linked to lysosomal dysfunction and accumulation (Martins et al. 2013). To determine lysosomal accumulation as arbitrary units (a.u.), the NRU survival rate was normalized to the average MTT and CVS survival rates using the function *w*(*x*, *y*, *z*), as previously described (Martins et al. 2017).

$$w=\frac{1}{2}\left[\left(\frac{x}{y}\right)+\left(\frac{x}{z}\right)\right]$$

*x*, *y*, and *z* were the survival rates measured by NRU, CVS, and MTT assays, respectively.

### 2.5 Immunostaining

After treatment of HaCaT cells with 20 µM triterpenoids or 0.25% (v/v) DMSO for 24 h at 37ºC, slides were washed twice in phosphate‐buffered saline (PBS) and fixed in 4.0% (w/v) formaldehyde in PBS for 15 min at 4ºC, followed by incubation in PBS containing 5.0% (w/v) of bovine serum albumin and 0.3% (v/v) Triton X‐100 for 60 min at room temperature. Next, we incubated the slides with primary monoclonal antibodies against LC3B, CTSB, and cytochrome c oxidase complex IV (COXIV) according to the manufacturer's instructions, followed by incubation with goat Alexa fluor‐coupled antibodies against rabbit IgG or mouse IgG. We analyzed the DAPI‐counterstained slides using a confocal microscope (Zeiss Axiovert 200 LSM 510 Laser, Carl Zeiss, Jena, Germany) equipped with a Plan‐APOCHROMAT 63X/1.40 oil DIC M27 objective (Zeiss, Carl Zeiss, Jena, Germany). As previously described, we determined the overlap between these proteins (Martins et al. 2015).

We analyzed the slides using filter sets that provide excitation of 364, 488, 543 and 633 nm with emission bandpass (BP) of 437–490 nm, 515–534 nm, 565–640 and 651–704 nm to detect the fluorescence of DAPI, Alexa Fluor 488 (A‐11034 or A‐11001), LTR, and MTDR or Alexa Fluor 633 (A‐21070 or A‐21050), respectively. Alternatively, we measured LC3B and COXIV‐related fluorescence using flow cytometry (BD FACS Verse). We collected at least 20,000 events in each analysis and analyzed the data using FlowJo Software Version 10.1.

### 2.6 Detection of Acidic Vesicular Organelles

We treated HaCaT with 20 µM triterpenoids or 0.25% (v/v) DMSO for 24 h. After incubating live cells with 1.0 µg/mL AO for 10 min at 37°C, we immediately visualized AO‐labeled acidic vacuoles (Shin et al. 2012) under an inverted epifluorescence microscope (Zeiss Axiovert 200, Germany) using an excitation BP of 450–490 nm with an emission long pass (LP) of 515 nm.

### 2.7 Citrate Synthase Assay

Citrate synthase (CS) is a crucial enzyme in the citric acid cycle, and its activity can serve as a marker for mitochondrial content and mitophagy efficiency (Martins et al. 2015). For monitoring CS activity, HaCaT cells were treated with 2 µM CCCP (Sigma‐Aldrich), 0.25% (v/v) DMSO, or 20 µM triterpenoids for 6 h. They were then rinsed with PBS and lysed using 0.25% (v/v) Triton X‐100 with inhibitors. After centrifugation to remove debris, the CS activity was determined, normalized to total protein, as described by Martins et al. (2015). Bafilomycin A1 treatment was used to monitor mitophagy functionality in terms of CS activity. It inhibits the activity of vacuolar‐type H^+^ ATPases (V‐ATPases), which are responsible for maintaining the acidic environment (pH ~4.5) of lysosomes (Yoshimori et al. 1991). Consequently, it leads to the alkalization of the lysosomal lumen which results in the loss of lysosomal degradative capacity of hydrolases like cathepsins.

### 2.8 Measurement of Cathepsin B and L Activities

We treated HaCaT with 20 µM triterpenoids or DMSO (0.25% v/v) for the indicated time, and we evaluated the activity of the lysosomal enzymes cathepsins B (CTSB) and L (CTSL) by a fluorometric assay (BioVision Inc.). Adherent cells were collected, sedimented by centrifugation, and lysed using a cold buffer (50–100 µL). The lysate was then incubated on ice for 10 min. Following this, we separated the cytosolic fraction from the remaining cell debris by centrifuging the mixture for 5 min at 10,000*g* at 4°C. Remained‐cell debris (e.g., containing the lysosomal fraction) was further lysed with more than 50–100 µL of chilled buffer, followed by freezing/thawing cycles (3×). After sedimentation of the extracted lysates (10,000*g* for 10 min at 4°C) and quantification by the Bradford assay (Bio‐Rad Laboratories), we employed 15–30 µg of protein per sample for enzymatic assays, according to the manufacturer's instructions. The enzyme activity was then normalized to total protein and represented as arbitrary units (a.u.) relative to the control condition (DMSO).

### 2.9 Cytosolic Cathepsin B by ELISA

Following treatment of HaCaT cells with 20 µM triterpenoids or DMSO (0.25% v/v) for 6 h, we assessed the quantity of cytosolic CTSB using an ELISA assay (R&D Systems DY2176). Briefly, we isolated cytosolic extracts as described above. After centrifugation of these cytosolic extracts for 10 min at 4°C and 10,000*g*, 10–15 µg of protein was used for immunodetection of CTSB, following normalization by total protein quantified using the Pierce Detergent Compatible Bradford assay (Thermo Scientific, 23246) and expressed as µg/mL.

### 2.10 Cell Clonogenic Assay

After 24 h of treatment with 20 µM triterpenoids or 0.25 (v/v) DMSO, we seeded HaCaT cells at an appropriate dilution to form colonies within 1 week, as previously described (Martins et al. 2017). After fixation with a 50% (v/v) ethanol solution, HaCaT cells were stained with a 0.02% (w/v) CV solution, washed twice, and counted using an inverted microscope equipped with transmitted light (Zeiss Axiovert 200, Germany). They were then imaged using ImageJ software.

### 2.11 Immunoblotting

We treated HaCaT with 20 µM triterpenoids or 0.25% (v/v) DMSO for 6 h. Cells were collected, centrifuged, lysed, and a western blot was performed as described (Martins et al. 2015). After incubating with primary anti‐PRKN, anti‐GAPDH antibodies, and secondary antibodies (anti‐rabbit HRP from KPL), the membranes were washed with PBS containing 0.1% (v/v) Tween 20 and with PBS alone. They were then incubated with ECL Prime Western Blotting Detection Reagent (GE Healthcare) and exposed to X‐ray films. The resulting images were analyzed using ImageJ software (National Institutes of Health, Bethesda), and the results were normalized to the intensities of the GAPDH bands.

### 2.12 Mitochondrial Function and Mass

Rh123 was used to monitor mitochondrial transmembrane potential (ΔΨm), and MTG and MTR were employed to measure cellular mitochondrial content, as previously published (Martins et al. 2019). According to the manufacturer's instructions, the following fluorescent probes were used to examine mitochondrial function (Galluzzi et al. 2007). Unlike Rh123 and MTR, MTG is a cell‐permeant, mitochondrial‐specific dye that becomes fluorescent only upon sequestration by mitochondria (Pendergrass, Wolf, and Poot 2004). We stained HaCaT with MTG, MTR, or Rh123, then washed and treated with 20 µM triterpenoids or 0.25% (v/v) DMSO. Next, we determined the fluorescence emission of at least 20,000 events using flow cytometry (BD FACS Verse) and analyzed the data with FlowJo software.

### 2.13 Assessing Apoptosis by Flow Cytometry With Annexin V/PI Labeling

We treated HaCaT with 20 µM triterpenoids or 0.25% (v/v) DMSO for 24 h. After the treatment, HaCaT cells were collected by trypsinization using 0.25% (w/v) EDTA–trypsin (Gibco, 25200056), centrifuged at 300*g* for 5 min, and washed twice with cold PBS before being resuspended in 500 μL of binding buffer. Subsequently, 5 μL of annexin V‐FITC (from Sigma‐Aldrich, APOAF kit) and 5 μL of PI were added. The samples were then incubated at room temperature in the dark for 10 min. A pool of treated cells was utilized to prepare control samples for establishing parameters during flow cytometry analysis in cell sorting. These control samples were created using either annexin V (AV) or PI alone.

AV binds to phosphatidylserine, which is externalized on the cell surface during the early stages of apoptosis. In contrast, PI is a DNA‐binding dye that stains cells with compromised membrane integrity, indicating late apoptosis or necrosis. We collected at least 20,000 events for cytofluorometric analysis using a BD FACS Verse and analyzed the data with FlowJo software. To distinguish between apoptotic and necrotic cells, we plotted the treated cells in a scatterplot and analyzed their distribution based on their PI (FL3) and AV (FL1) fluorescence. The cell subpopulations gated in the Q3 quadrant, PI (+)/AV (−), were further analyzed using a light scatter analysis based on 90° side scatter (SSC) and forward low‐angle light scatter (FSC) to distinguish between apoptosis (Q3), necrosis (Q4), and AACD (Q2).

### 2.14 MitoTracker Green FM/PI Double‐Labeled Flow Cytometry

We treated HaCaT with 20 µM triterpenoids or 0.25% (v/v) DMSO for 24 h (T1) or after 24 h posttreatment (T2). After treatment, the supernatant containing HaCaT cells was collected and stored on ice. Adherent HaCaT cells were then harvested by trypsinization using 0.25% (w/v) EDTA–trypsin (Gibco, 25200056) and transferred to a Falcon tube containing the supernatant. Following centrifugation at 300*g* for 5 min, the cell pellet was resuspended in 1 mL of 0.05% (w/v) EDTA–PBS. From this mixture, 100 µL of each sample was collected to prepare a pooled cell sample for staining control. Subsequently, 10 µL of 10 µM MTG and 10 µL of 0.5 mg/mL PI were added. The samples were incubated for 10 min at room temperature, followed by a second centrifugation at 300*g* for 5 min at 4°C. Finally, the cells were resuspended in 300 µL of 0.05% (w/v) EDTA–PBS for immediate analysis by flow cytometry. Additionally, a pool of treated cells was used to prepare control samples for establishing parameters during flow cytometry analysis in cell sorting, which were created using only MTG or PI. We collected at least 30,000 events for cytofluorometric analysis using a BD FACS Verse and analyzed the data with FlowJo software. We performed a light scatter analysis to evaluate further granularity and vacuolization, which are indicative of autophagy activation, using SSC and FSC. We evaluated vacuolated and granulated cells (Q2) in terms of mitochondrial mass.

### 2.15 Liposomes and Carboxyfluorescein Leakage

We determined the membrane permeabilization mediated by BA or UA, as indicated by the leakage of unilamellar liposomes containing trapped CF (Sigma‐Aldrich). We treated liposomes with 20–100 µM triterpenoids or 0.25%–1.25% (v/v) DMSO for 30 min and then measured CF release using a TECAN plate reader. We calculated the rates of CF release as a percentage of total trapped CF release (Ft) relative to total membrane disruption using 0.2% (v/v) Triton X‐100, as described by Rodrigues et al. (2016).

### 2.16 Giant Unilamellar Vesicles as a Membrane Model

We prepared the giant unilamellar vesicles (GUVs) containing POPC bilayer‐based GUVs, specifically 2‐oleoyl‐1‐palmitoyl‐sn‐glycero‐3‐phosphocholine (C16:0, 18:1) or POPC: cardiolipin (CL) (8 mol: 2 mol), using the electroformation method as described (Angelova and Dimitrov 1986). We added triterpenoids solubilized in DMSO at 4 mg/mL or 100% (v/v) DMSO to the vesicles to achieve a final concentration of 100 µM or 1.25% (v/v), respectively, and immediately placed them in the observation chamber. We examined the treated GUVs under an inverted microscope, Axiovert 200 (Zeiss). Images were captured using an AxioCam HSm digital camera (Zeiss) and processed using ImageJ Software (National Institutes of Health, Bethesda).

### 2.17 Erythrocyte as a Minimum Cell

Following the methodology of Gao et al. (2014), we used erythrocytes from fresh human blood, with ethical approval from Anhanguera University of São Paulo (CAAE 91569018.3.0000.5493). The erythrocytes were diluted, counted, and resuspended in PBS. We seeded them (4 × 10^6^) in a 96‐well round‐bottom plate and treated them with 100 µM triterpenoids or 1.25% (v/v) DMSO at room temperature for 24 h. Next, we sedimented the treated erythrocytes and fixed them in 4% (w/v) formaldehyde in PBS. We washed them in a 50% (v/v) ethanol solution and stained them with a 0.02% (w/v) CV solution for 5 min. After mounting the slides with 90% (v/v) glycerol in PBS, we proceeded as described above.

### 2.18 Statistical Analysis

All statistical analyses were performed using IBM SPSS Statistics version 20. Pearson's coefficient was applied to assess the strength of linear correlations. Variance among sample groups was first examined using Levene's Test, and comparative statistics were conducted using one‐way ANOVA with either the Dunnett T3 or Bonferroni post hoc test, depending on the homogeneity of variance. A *t*‐test for independent samples was used for a pair‐wise comparison for parametric data. Outliers were managed using a two‐step approach. First, data points with kurtosis values exceeding 5.0 were flagged as potential outliers, as the kurtosis test is a robust and versatile method for detecting outliers in small samples (Iacobucci et al. 2025; Livesey 2007). These flagged values were investigated further to assess their impact on the results. Second, outliers identified using the interquartile range (IQR) method were excluded only if their removal altered the mean value by more than 10%. This combined approach ensures a rigorous and systematic handling of extreme values, minimizing the risk of erroneous conclusions or misinterpretation of the data (Iacobucci et al. 2025). Data from at least three independent experiments performed in triplicate were presented as means ± standard errors. Statistical significance was defined as *p* values below 0.05, with significance levels denoted by asterisks above the bars in graphs, compared to the control condition DMSO: *p < 0.05, **p < 0.01, and ***p < 0.001.

## 3 Results

### 3.1 Impact of Triterpenoids on Cell Viability and Mitochondrial Function

**Figure 1:**
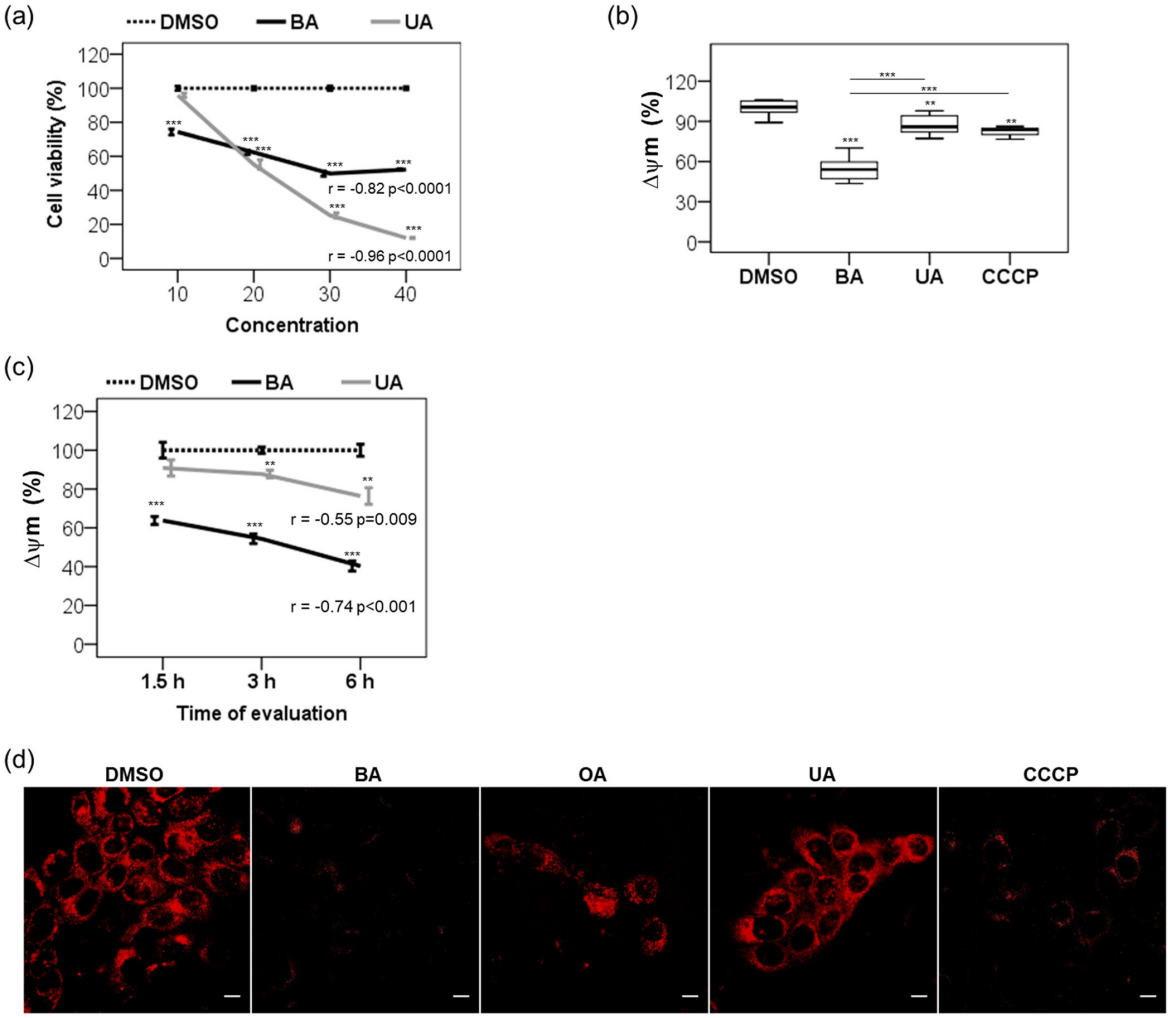
Comparative evaluation of triterpenoid effects in nonmalignant human keratinocytes. (a) Viability of HaCaT cells after varying BA or UA concentrations (10–40 µM) for 24 h, analyzed by the MTT assay at 48 h. DMSO levels ranged from 0.125% to 0.50% (v/v). (b) Rh123 flow cytometry was used to measure the ΔΨm reduction after a 3‐h treatment with 20 µM triterpenoids or 2 µM CCCP, compared to 0.25% (v/v) DMSO. (c) Time‐dependent ΔΨm changes following 20 µM triterpenoids or 0.25% (v/v) DMSO. (d) Microscopy analysis of HaCaT following staining for mitochondria with MitoTracker Red CM‐H_2_XRos (red) and treatment for 6 h with 0.25% (v/v) DMSO, 20 µM triterpenoids, or 2 µM CCCP. Results are from at least three independent experiments (*n* = 3), and we presented them as mean ± standard error. ANOVA post hoc test Dunnett T3 (a) or Bonferroni (b and c) was performed, and significance levels are indicated as **p < 0.01, ***p < 0.001. Asterisks above bars represented the statistical significance compared to the DMSO control. Scale bar: 10 µm (d).

BA and UA share similar physicochemical properties (Supporting Information S1: Table 1) and significantly influence the viability of nonmalignant keratinocytes (Figure 1). The 20 µM concentration used in this study aligns with values reported in the literature (X. Chen et al. 2020; Liu et al. 2019; Potze et al. 2014; Sun et al. 2021; Yang et al. 2012). At this dose, both compounds exhibited comparable reductions in cell survival (*p* = 0.087). However, at 40 µM, UA reduced viability to 12%, whereas BA only reduced it to 52% (*p* < 0.0001; Figure 1a).

After 3 h of treatment, BA induced a greater mitochondrial membrane potential (ΔΨm) than UA and the mitochondrial uncoupler CCCP (*p* < 0.000001; Figure 1b). Over time, BA caused a persistent decline in ΔΨm (*r* = –0.74, *p* < 0.001; Figure 1c). This observation was confirmed via confocal microscopy with MTR staining (Figure 1d). Only BA, oleanolic acid (OA), and CCCP significantly reduced MTR signal intensity after 6 h. These findings are consistent with prior studies (Duval et al. 2008; Martins et al. 2015), indicating that BA induces mitochondrial dysfunction, while UA preserves mitochondrial function under the same conditions, suggesting divergent mechanisms of action.

### 3.2 Modulation of Mitophagy by UA and BA

**Figure 2:**
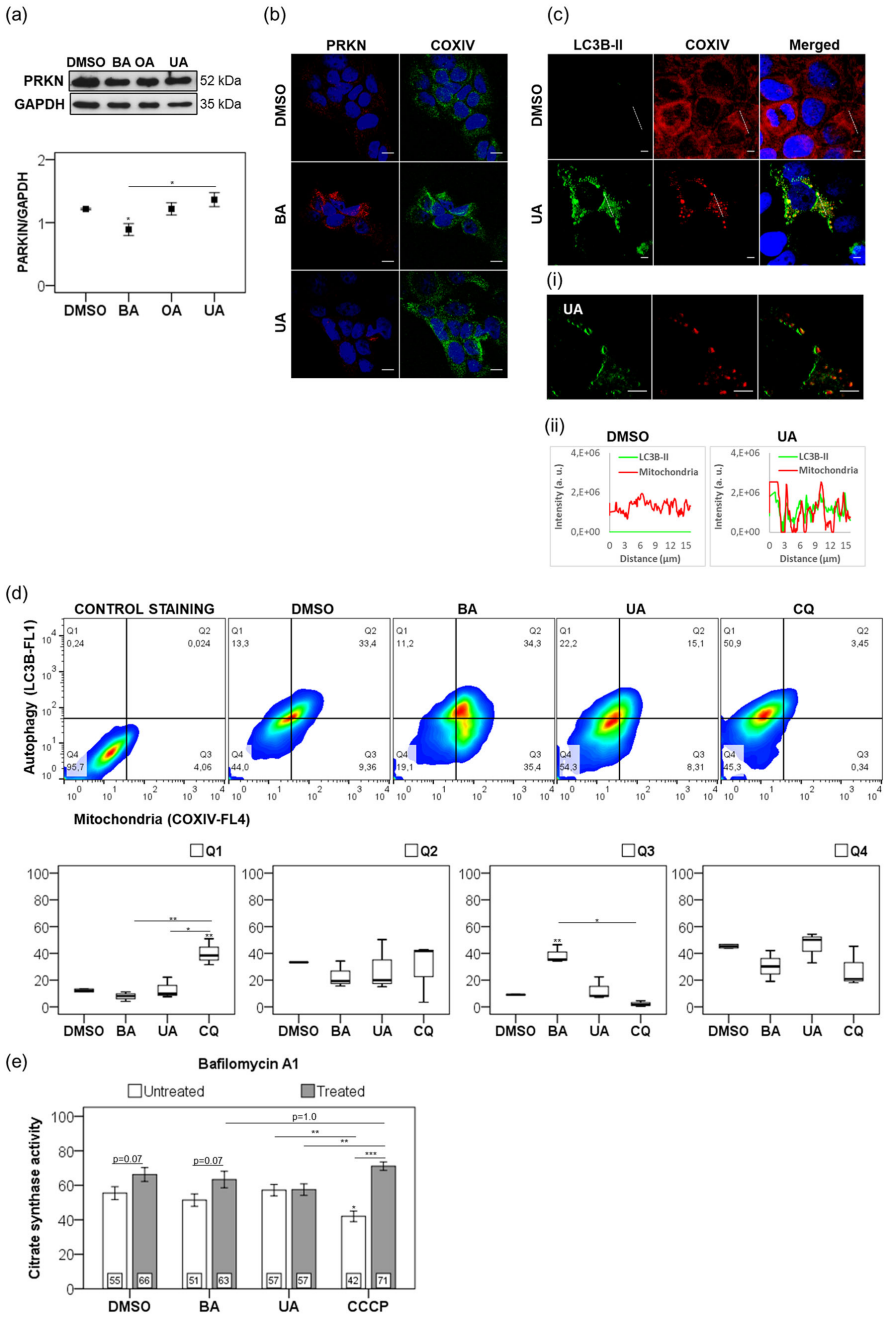
Modulation of mitophagy in nonmalignant keratinocytes. (a) PRKN expression normalized to GAPDH in HaCaT treated with 0.25% (v/v) DMSO, BA, OA, or UA (20 µM) for 6 h. (b) Confocal microscopy following COXIV (green) and PRKN (red) immunostaining after a 6‐h treatment with 20 µM triterpenoids or 0.25% (v/v) DMSO. (c) LC3B‐II (green) and COXIV (red) immunostaining after 24 h with UA (20 µM), generating LC3B‐II/COXIV fluorescence profiles. (i) Micrographs of UA‐treated cells (lined area) showing recruitment of LC3B‐II (green) to mitochondria (COXIV in red). (ii) Fluorescence plot profiles represented line scans of mitochondria and LC3B‐II. (d) LC3B‐II or mitochondrial mass (COXIV) immunostaining was analyzed by cytofluorometry after 24 h. We used chloroquine (60 µM) as a standardized condition to induce/inhibit autophagy (Klionsky et al. 2021). (e) Citrate synthase activity assay with or without bafilomycin A1 (10 nM) within 6 h. Results are from at least three independent experiments (*n* = 3), and we presented them as mean ± standard error. ANOVA post hoc test Dunnett T3 (a and d‐Q1) or Bonferroni (d‐Q2–Q4 and e) was performed according to the groups (DMSO, BA, OA, UA, or CQ), and the *t*‐test for independent samples was used for a pair‐wise comparison concerning bafilomycin A1 treatment. Significance levels are indicated as *p < 0.05, **p < 0.01, ***p < 0.001. Asterisks above bars represented the statistical significance compared to the DMSO control. Scale bars: 10 µm (b and c).

The Parkin (PRKN) axis plays a critical role in mitophagy by selectively clearing damaged mitochondria (Narendra et al. 2008). Treatment with UA resulted in a modest 12% increase in total PRKN expression versus DMSO (*p* = 0.704), whereas BA significantly enhanced PRKN recruitment to mitochondria, coinciding with loss of ΔΨm—Figures 1c,d and 2a,b. This effect resembled that induced by OA, a known mitophagy inducer in both malignant and nonmalignant cells (Martins et al. 2015; Castrejón‐Jiménez et al. 2019).

UA induced LC3B‐II recruitment to mitochondria (COXIV‐positive), suggesting efficient mitophagic flux (Figure 2c(i)). Fluorescence intensity profiles confirmed colocalization of LC3B‐II with mitochondria in UA‐treated cells (Figure 2c(ii)). Flow cytometry revealed that both BA and UA—but not CQ—increased LC3B‐II–COXIV colocalization in a subpopulation of cells (Q2 quadrant, Figure 2d). Notably, only UA increased LC3B levels without a corresponding rise in mitochondrial mass (Q1), further supporting efficient mitophagy. Therefore, BA caused significant mitochondrial accumulation, indicating impaired mitophagy, whereas UA promoted efficient mitophagy. Previous findings support this observation (Martins et al. 2015; Castrejón‐Jiménez et al. 2019).

CS activity, a marker of mitochondrial content and mitophagy efficiency, supported these findings. BA impaired mitophagy, maintaining high CS activity even in the presence of lysosomal inhibition by bafilomycin A1 (Figure 2e), consistent with previous results (Martins et al. 2015). In contrast, CCCP significantly reduced CS activity, which was reversed by bafilomycin A1, reflecting expected mitophagic degradation. UA induced only modest mitophagy, as evidenced by stable CS activity and limited ΔΨm loss (Figures 1b and 2e). Altogether, these data suggest that BA promotes mitochondrial damage and impaired mitophagy, whereas UA preserves ΔΨm and supports functional mitophagy in nonmalignant keratinocytes.

### 3.3 Triterpenoids' Effect on Lysosomal Function in Nonmalignant Human Keratinocytes

**Figure 3:**
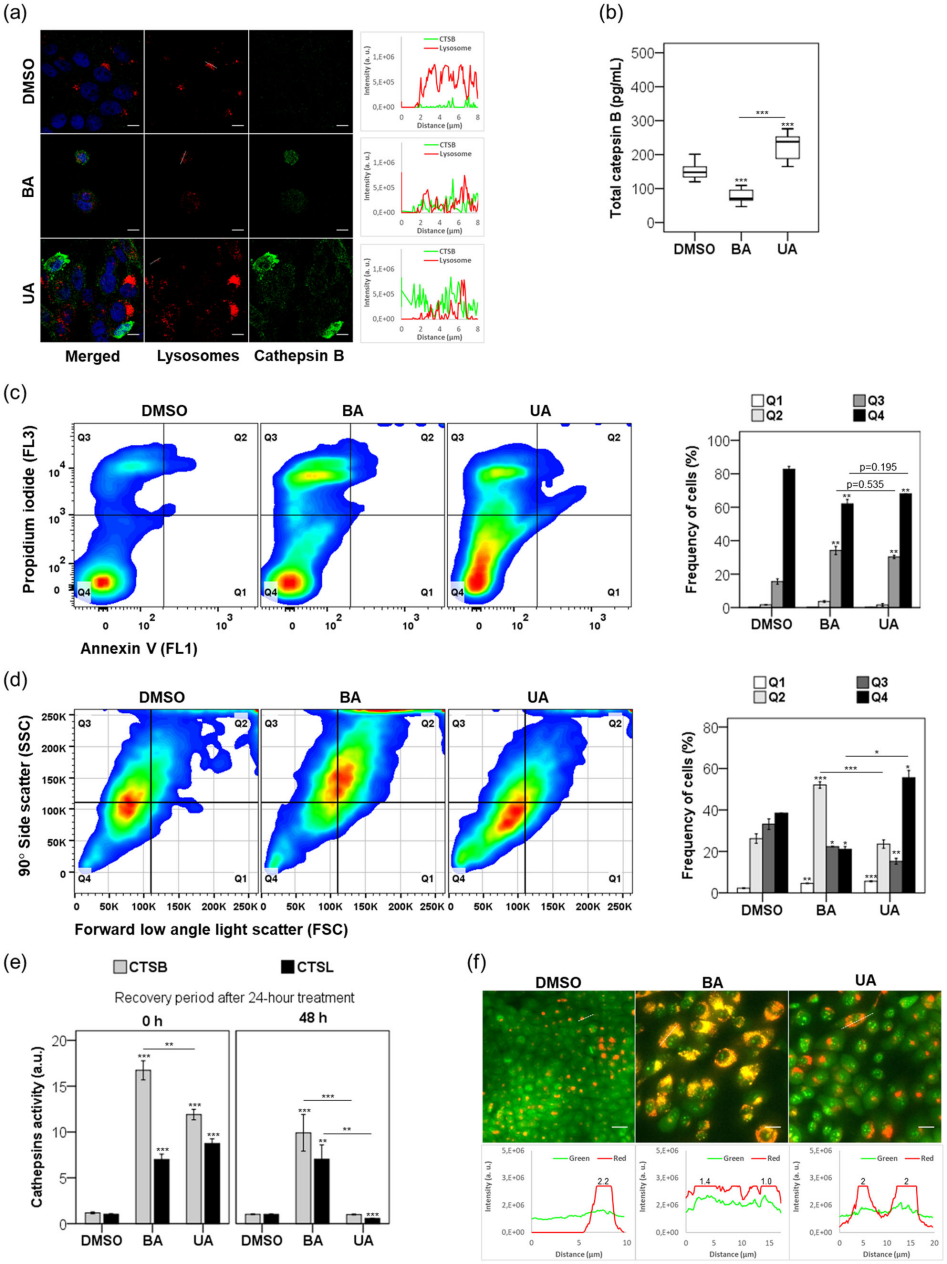
UA induces lysosomal membrane permeabilization and cell death in HaCaT cells. We treated HaCaT with 20 µM triterpenoids or 0.25% (v/v) DMSO for 6 h and (a) quantified total cytosolic CTSB by ELISA assay and (b) immunostaining for CTSB (green) and lysosomes loaded with LysoTracker Red DND‐99 (red). At right, fluorescence plot profiles represented line scans of CTSB and LTR‐loaded lysosomes. (c) After 24‐h treatment with 0.25% (v/v) DMSO and 20 µM triterpenoids, cells were stained with annexin V and propidium iodide and gated according to (FL1) and (FL3) fluorescence following FACS. A representative scatterplot displays the subpopulations Q1, Q2, Q3, and Q4 (upper panel), while a graph illustrates the frequency distribution of these subpopulations (bottom panel). (d) Gated AV^−^/PI^+^ (Q3) cells plotted according to 90° Side scatter (SSC) and forward low‐angle light scatter (FSC) parameters and analyzed through scatterplot distribution (upper panel). We represented the frequency of cell subpopulations with bars (bottom panel). (e) Activity of CTSB and CTSL in lysosomal fraction by fluorometric assay after treatment with 20 µM triterpenoids or 0.25% (v/v) DMSO at indicative times. (f) After a 48‐h recovery period, HaCaT cells treated with 0.50% (v/v) DMSO or 40 µM triterpenoids were stained with acridine orange (AO) and imaged. At the bottom, fluorescence plot profiles represented line scans of the lysosomotropic dye AO's green and red fluorescence intensity. Results are from at least three replicates and two independent experiments, presented as mean ± standard error. ANOVA post hoc test Dunnett T3 (b, d‐Q4, and e) or Bonferroni (c and d‐Q1–Q3) were performed, and significance levels are indicated as *p < 0.05, **p < 0.01, ***p < 0.001. Asterisks above bars represented the statistical significance compared to the DMSO control. Scale bars: 10 μm (a and f).

Within 6 h, BA and UA significantly reduced LysoTracker Red fluorescence in a subset of HaCaT cells (Figure 3a, lined region), indicating loss of lysosomal membrane permeabilization (LMP). UA markedly increased cytosolic CTSB, while BA caused only a modest elevation (Figure 3a, line scans), suggesting differential impacts on lysosomal integrity.

An ELISA assay confirmed these observations: UA significantly increased cytosolic CTSB levels by 50% compared to DMSO (*p* < 0.00001), whereas BA's effect was less pronounced (Figure 3b). Flow cytometry analysis following AV and PI staining revealed that BA and UA both increased the proportion of cells in late apoptosis/necrosis (Q3 quadrant)—34% and 30%, respectively—compared to DMSO (Figure 3c; *p* < 0.01).

Light scatter analysis of Q3‐gated cells further demonstrated that BA caused notable increases in vacuolization and granularity (52%, *p* = 0.002), indicative of AACD. In contrast, UA‐treated cells exhibited significantly lower granularity (24%, *p* < 0.0001). Interestingly, the frequency of PI(+)/AV(−) cells (Q4), potentially representing necrosis, was higher with UA (56%) than with BA (21%), *p* = 0.011—Figure 3d.

After a 24‐h treatment followed by a 48‐h recovery period, we assessed lysosomal functionality by measuring CTSB and CTSL activity (Figure 3e). While both triterpenoids increased cathepsin activity at 24 h, only the UA‐treated cells restored CTSB and CTSL activity to baseline levels after recovery. In contrast, BA induced persistent lysosomal dysfunction, characterized by elevated cathepsin activity and the accumulation of acidic vesicular organelles (AVOs), as visualized via AO staining (Figure 3f).

At 40 μM, UA‐treated cells exhibited intense red fluorescence, indicating the presence of acidic, functional lysosomes. In contrast, BA‐treated cells exhibited mixed orange–red fluorescence, indicating alkalinization and disruption of lysosomal pH. Intensity profile plots confirmed that UA maintained a high red‐to‐green ratio (~2.0), while BA‐treated cells displayed heterogeneous fluorescence and a lower red/green ratio (~1.0–1.4)—Figure 3f.

These results indicate that while both BA and UA impact lysosomal integrity, UA induces reversible lysosomal damage with full recovery, whereas BA causes sustained lysosomal dysfunction associated with AACD. These findings align with prior studies in both malignant and nonmalignant models (Martins et al. 2015, 2017; Lena et al. 2009; Shin et al. 2012; Navanesan et al. 2017).

### 3.4 Long‐Term Triterpenoids' Effects on Nonmalignant Human Keratinocytes

**Figure 4:**
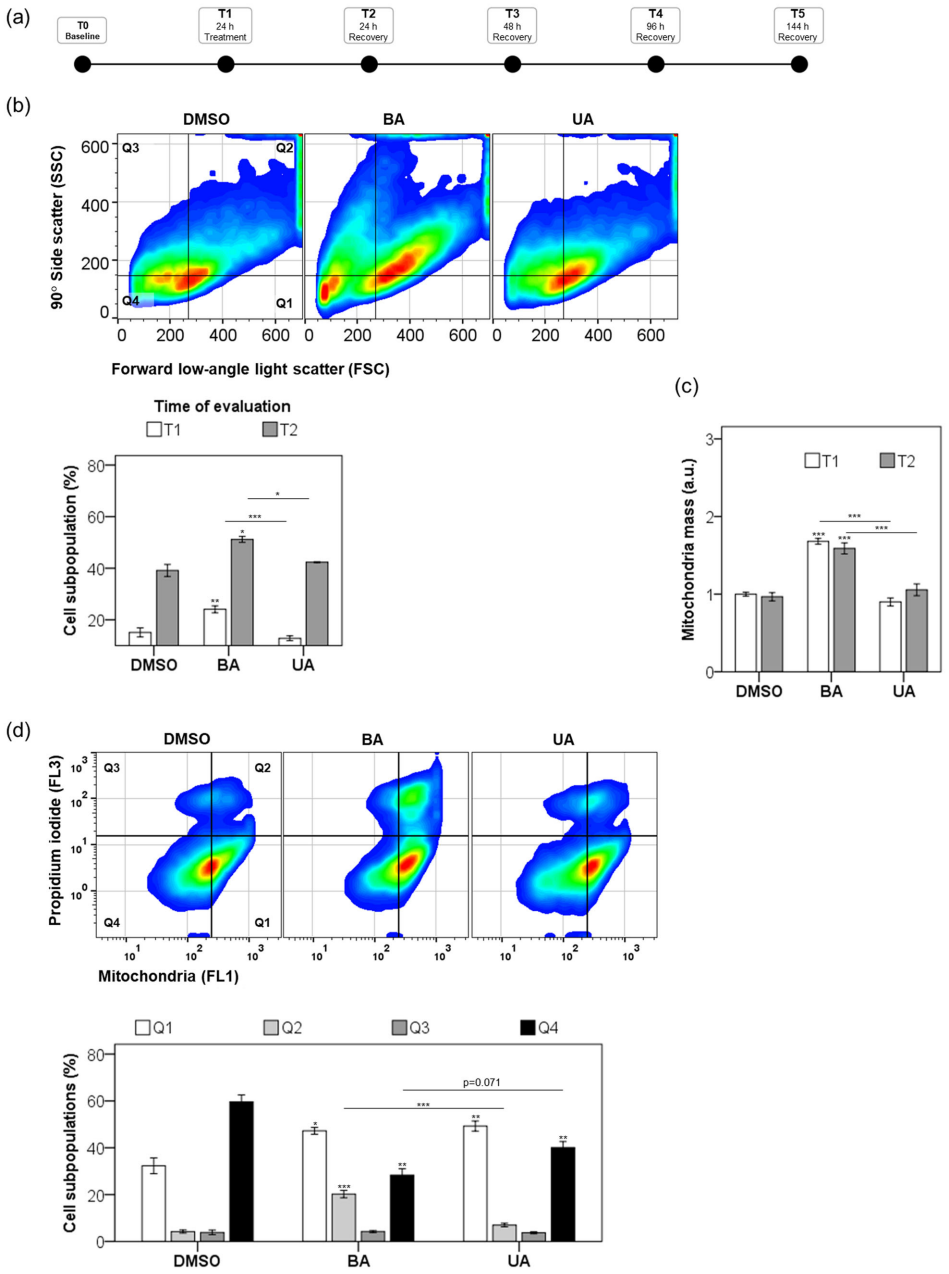
Assessment of mitochondrial mass and subpopulation analysis following triterpenoid treatment. (a) The experimental timeline consisted of distinct phases. Following a 24‐h treatment (T1), HaCaT cells were washed and chased for additional periods of 24 h (T2) to 144 h (T5). (b) After 24‐h treatment with 0.25% (v/v) DMSO and 20 µM triterpenoids, cells were stained with propidium iodide and MitoTracker Green FM (mitochondria mass) and gated based on 90° side scatter (SSC) and forward low‐angle light scatter (FSC) parameters. A representative scatterplot from cells at T2 shows the subpopulations [Q1, Q2, Q3, and Q4] (upper panel). Bottom panel: we represented the frequency of cell subpopulations from Q2 at T1 and T2 time points in bars. (c) Cell subpopulations from Q2 were analyzed regarding the median fluorescence of mitochondria stained with MitoTracker Green FM (MTG). In bars, the mitochondrial mass of treated cells was represented as a fold change compared to the DMSO control. (d) Representative scatterplot showing the subpopulations [Q1, Q2, Q3, and Q4] from cells gated based on propidium iodide (FL3) and MTG‐loaded mitochondria (FL1) and a graph representing the frequency of these subpopulations (bottom panel). Results are from at least three independent experiments (*n* = 3) and expressed as mean ± standard error. ANOVA post hoc test Bonferroni (b‐T1, c‐T2, and d) or Dunnett T3 (b‐T2 and c‐T1) were performed, and significance levels are indicated as *p < 0.05, **p < 0.01, ***p < 0.001. Asterisks above bars represented the statistical significance compared to the DMSO control.

UA and BA elicited distinct long‐term responses in HaCaT keratinocytes (Figure 4a). After 24 h of treatment, BA significantly increased cell size and granularity (24%) compared to UA (12%) (*p* < 0.00001; Figure 4b). Unlike UA, BA‐treated cells showed persistent vacuolization and mitochondrial accumulation even after a recovery period (Figure 4c), correlating with sustained cell death (Figure 4d).

**Figure 5:**
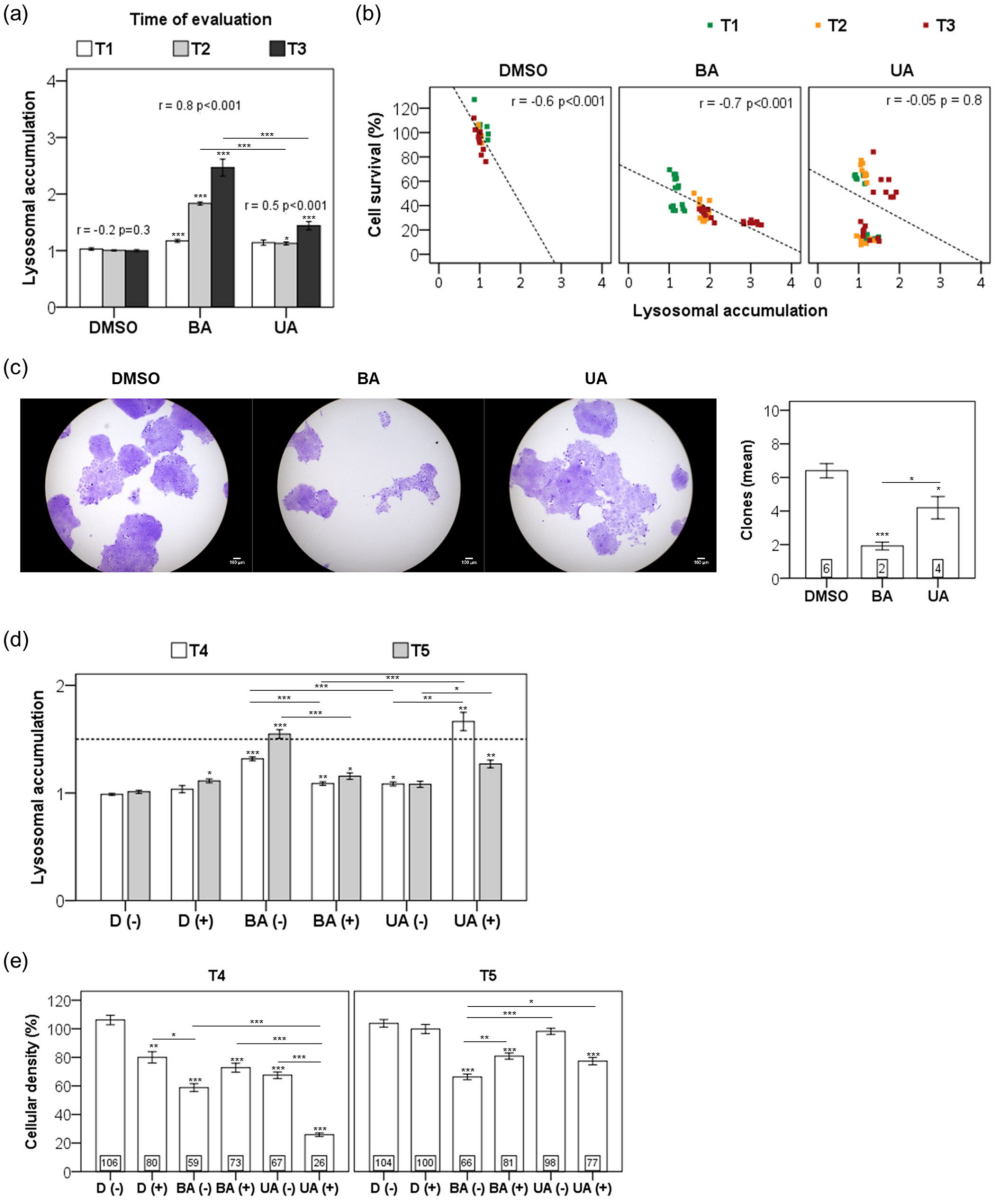
Assessment of long‐term effects of triterpenoids on nonmalignant cells. Following a 24‐h treatment (T1), HaCaT cells were washed and chased for additional periods of 24 h (T2) to 48 h (T3). HaCaT cells were treated with 20 µM triterpenoids or 0.25% (v/v) DMSO for 24 h, followed by cell survival analysis measured by the CVS assay and lysosomal accumulation, represented as arbitrary units (a.u.), at specific time points (T1, T2, and T3). We determined (a) the lysosomotropic vacuolization and its correlation with time of evaluation, and (b) cell survival determined by Pearson's coefficients. (c) Micrographs showing CV cytochemical staining of colonies 7 days after a 24‐h treatment with 20 µM triterpenoids or 0.25% (v/v) DMSO. On the right, the number of colonies is represented in bars. (d) HaCaT cells were treated with 30 µM triterpenoids or 0.50% (v/v) DMSO (referred to as D) in the presence (+) or absence (−) of 50 µM chloroquine (CQ) for 24 h. Following this 24‐h treatment, cells were washed and incubated for 96 (T4) or 144 h (T5). We represented the lysosomal accumulation in treated cells as a fold change in bars compared to the DMSO control. In (e), we represented the percentage of the cellular density measured by the CVS assay compared to the DMSO control. Results are from at least three independent experiments (*n* = 3) and expressed as mean ± standard error. ANOVA post hoc test Dunnett T3 (a‐T1, c, and d) or Bonferroni (a‐T2, a‐T3, and e) were performed, and significance levels are indicated as *p < 0.05, **p < 0.01, ***p < 0.001. Asterisks above bars represented the statistical significance compared to the DMSO control. Scale bars: 100 μm (c).

While UA‐treated cells restored lysosomal and mitochondrial function posttreatment, BA exposure resulted in ongoing dysfunction. Over time, lysosomotropic vacuolization was strongly correlated with BA exposure and cell demise (*r* = 0.8, *p* < 0.001; Figure 5a). In contrast, this correlation was absent for UA‐treated cells (*r* = −0.05, *p* = 0.8; Figure 5b).

Clonogenic assays revealed that BA significantly impaired cell proliferation (30% of DMSO; *p* < 0.0001), whereas UA preserved higher colony‐forming capacity (70% of DMSO; *p* = 0.039), ultimately reaching 98% of control density (*p* = 0.759)—Figure 5c,e. These findings suggest that UA supports recovery via cytoprotective autophagy, while BA leads to long‐lasting impairment.

Interestingly, CQ modulated these outcomes. CQ potentiated UA's cytotoxicity (61% decrease in viability; *p* < 0.0001), mimicking BA's effects. However, CQ mitigated BA's long‐term cytotoxicity by reducing lysosomal accumulation (Figure 5d) and improving survival rates (Figure 5e). These results suggest that CQ may enhance UA's therapeutic potential while attenuating BA's harmful effects on nonmalignant keratinocytes.

### 3.5 Membrane Permeability and Interaction With Model Membranes

**Figure 6:**
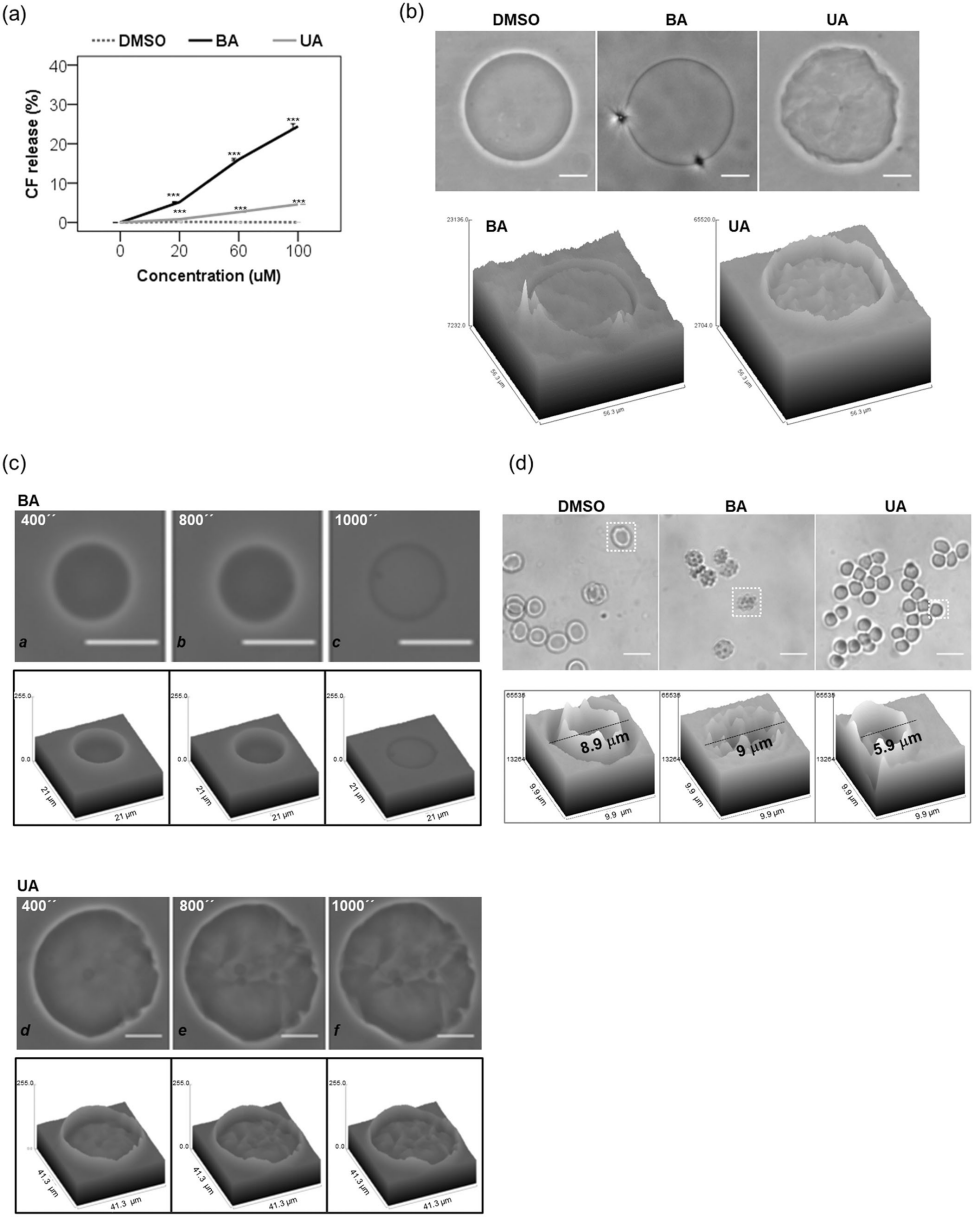
Membrane damage analysis by triterpenoids. (a) Cell membrane permeability analysis based on carboxyfluorescein (CF) release from unilamellar liposomes. Liposomes were treated with 20–100 µM triterpenoids or 0.25%–1.25% (v/v) DMSO for 30 min, and the CF release was measured. (b) Micrographs of giant unilamellar vesicles made of POPC to evaluate the membrane perturbation after treatment with 100 µM triterpenoids or 1.25% (v/v) DMSO over time (indicated in seconds in the left upper corner of each image). Surface plots of the depicted POPC membranes are highlighted for visualization (bottom panel). The displayed images are representative phase‐contrast images from three different experiments. (c) The micrographs depict giant unilamellar vesicles composed of POPC: CL. We captured these images to assess the membrane perturbation following treatment with 100 µM triterpenoids or 1.25% (v/v) DMSO. We indicated the time elapsed in seconds in the upper left corner of each image following treatment with BA or UA. The displayed images are representative phase‐contrast images from three different experiments. (d) These micrographs display erythrocytes after a 24‐h treatment with 100 µM triterpenoids or 1.25% (v/v) DMSO. The images showcase the morphological changes induced by the triterpenoid treatment. Surface plots of the depicted erythrocytes are highlighted in the bottom panel for visualization. Results from at least three independent experiments (*n* = 3), expressed as mean values ± standard error. ANOVA post hoc test Bonferroni (a) was performed, and significance levels are indicated as *p < 0.05, **p < 0.01, ***p < 0.001. Asterisks above the bars represent statistical significance compared to the DMSO control. Scale bars: 10 µm (b and c).

We explored the membrane‐disruptive effects of triterpenoids using protein‐free, mitochondria‐like membranes and human erythrocytes. BA caused significantly greater membrane disruption than UA, as indicated by CF leakage from unilamellar liposomes (Figure 6a). This finding aligns with previous reports, which show that BA increases membrane fluidity and permeability, particularly in mitochondria and lysosomes (Martins et al. 2017).

We further assessed how BA and UA (100 μM) interact with GUVs composed of POPC (2‐oleoyl‐1‐palmitoyl‐sn‐glycero‐3‐phosphocholine). BA strongly permeabilized POPC membranes, while UA induced only time‐dependent surface deformation without significant membrane leakage (Figure 6b).

Next, we evaluated GUVs composed of POPC and CL to mimic mitochondrial membranes (Figure 6c). BA increased membrane permeability, while UA caused only surface‐level changes without inducing rupture. These observations support the notion that BA disrupts membrane integrity more aggressively than UA, especially in CL‐enriched environments.

Despite UA's effects on simple lipid bilayers, it had minimal impact on more complex membranes such as human erythrocytes. UA reduced cell volume by 34% (consistent with Jilani et al. 2011), but did not cause noticeable changes in curvature or membrane structure. Conversely, BA induced echinocyte formation—small, spiked projections on the erythrocyte surface—indicating membrane perturbation and potential for eryptosis (Figure 6d).

Together, these results underscore the distinct interactions of BA and UA with cellular membranes. While BA permeabilizes membranes broadly and disrupts structural integrity, UA's membrane effects are subtler and more dependent on lipid composition. These differential membrane interactions may underlie their distinct mechanisms of inducing cell death, suggesting unique avenues for selective antitumor strategies.

### 3.6 Differential Antitumor Effects of Triterpenoids on Malignant Cell Lines

**Figure 7:**
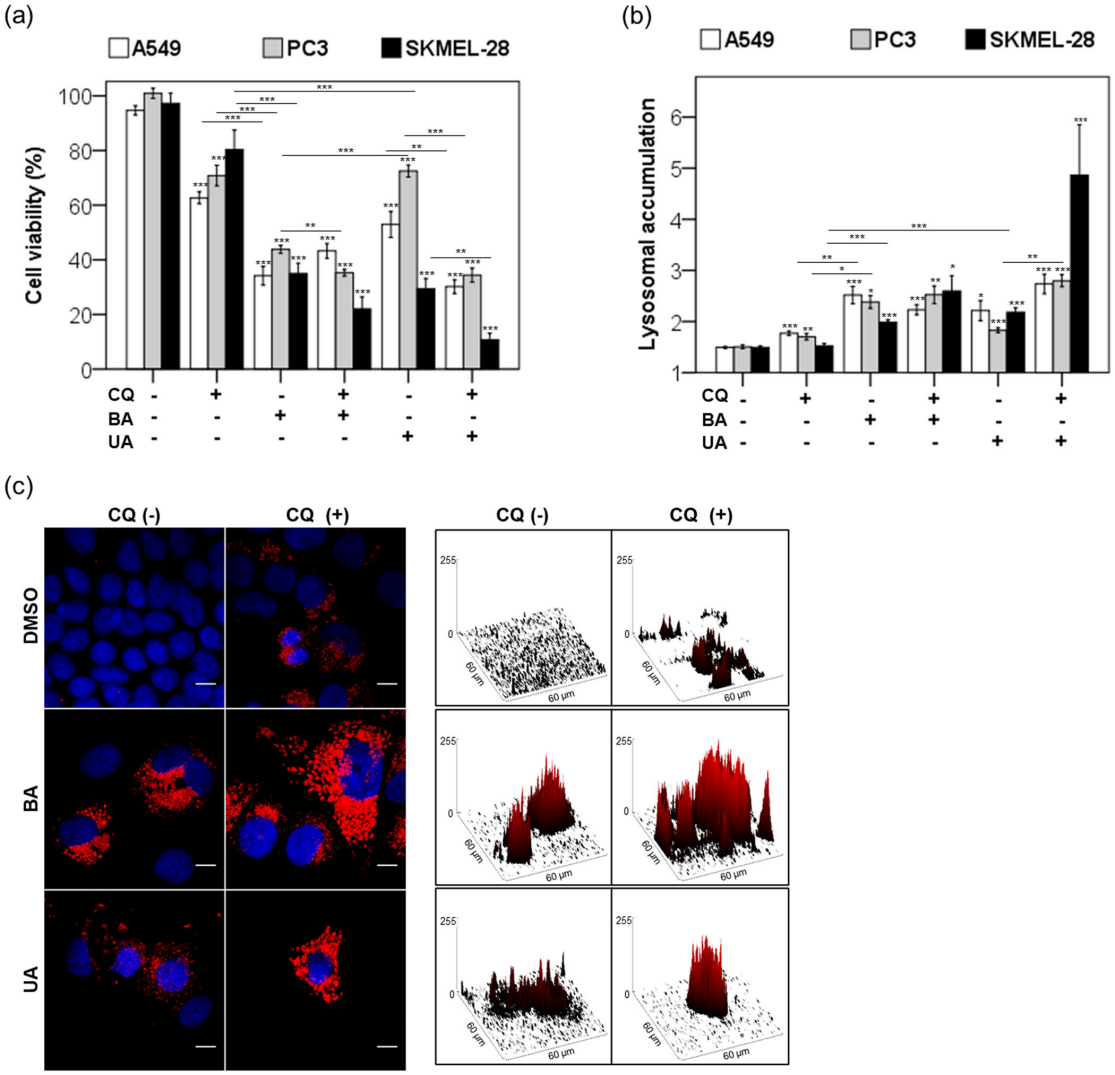
Effects of triterpenoids in human malignant cells. We treated human malignant cell lines (A549, PC3, MCF7, and SKMEL‐28) with 20 µM triterpenoids or 0.25% (v/v) DMSO in the presence (+) or absence (−) of 20 µM chloroquine (CQ) for 24 h: In (a), we represented the percentage of the cellular density measured by the MTT assay in comparison to the DMSO control after 48‐h following the 24‐h treatment. In (b), we represented the lysosomal accumulation of treated cells as a fold change in bars compared to the DMSO control. (c) After a 48‐h recovery period following a 24‐h treatment with 0.25% (v/v) DMSO or 20 µM triterpenoids, lysosomes of treated MCF7 cells were stained with LysoTracker Red DND‐99 (LTR) and imaged. Right panel: LTR intensity is represented by surface plots. Results from at least three independent experiments (*n* = 3) are expressed as mean values ± standard error. ANOVA post hoc test Dunnett T3 (a and b) was performed, and significance levels are indicated as *p < 0.05, **p < 0.01,***p < 0.001. Asterisks above the bars represent statistical significance compared to the DMSO control. Scales bar: 10 µm (c).

Although all seven malignant cell lines were tested—A549 (lung), HeLa (cervix), MES‐SA (uterine sarcoma), PC3 (prostate), MCF7 (breast), SKMEL‐25, and SKMEL‐28 (melanoma)—representative data from four tumor types (A549, PC3, MCF7, and SKMEL‐28) are presented graphically in Figure 7. Both cell viability and lysosomal accumulation data for HeLa, MES‐SA, and SKMEL‐25 cell lines are available in Supporting Information S2: Table 2.

BA markedly reduced the viability of all seven malignant cell lines tested, with survival rates ranging from 29.8% to 51.9% (*p* < 0.00001; Supporting Information S2: Table 2). In contrast, UA was less effective than BA, particularly in PC3 cells, which retained 72.5% viability (*p *< 0.00001; Figure 7a). These findings are consistent with previous studies, which have shown variable cytotoxicity of BA and UA depending on the cell line and exposure time, with IC_50_ values ranging from 7 to 146 μM and 11.4 to 77.7 μM, respectively (National Center for Biotechnology Information 2024a, 2024b).

BA's cytotoxicity correlated with increased lysosomal accumulation, particularly in PC3 (*p* = 0.05; Supporting Information S2: Table 2). However, SKMEL‐28 cells, despite exhibiting lower lysosomal accumulation than A549, showed similarly reduced viability (~35%; Figure 7b), suggesting that lysosomal stress is not the sole driver of cell death.

Cotreatment with CQ enhanced the cytotoxic effects of both triterpenoids in a cell‐type‐dependent manner (Figure 7a). In SKMEL‐28, UA combined with CQ led to greater lysosomal accumulation than BA alone (Figure 7b). In MCF7 cells, BA—but not UA—consistently triggered acidic vacuole accumulation regardless of CQ cotreatment (Figure 7c). Interestingly, while CQ exacerbated BA‐induced stress in SKMEL‐28 (Figure 7a,b), it improved long‐term recovery of BA‐treated nonmalignant keratinocytes by reducing lysosomal dysfunction (Figure 5d,e), highlighting its potential for selective combination strategies.

## 4 Discussion

This study reveals how structurally similar triterpenoids, UA and BA, engage distinct pathways to modulate autophagy, organelle stability, and cell fate in both malignant and nonmalignant human cells. Emphasis was placed on nonmalignant keratinocytes, a model of high clinical relevance in skin biology and treatment‐associated toxicity.

Both triterpenoids impaired HaCaT cell viability, but through divergent pathways. BA‐driven mitochondrial stress likely activated PRKN‐mediated mitophagy and persistent lysosomal dysfunction, while concurrently causing a prolonged lysosomal–mitochondrial stress. These BA effects ultimately compromised protective autophagy and cell recovery, which may culminate in the accumulation of ROS and death, as evidenced in prior studies (Coricovac et al. 2021; Lewinska et al. 2017; Martins et al. 2015, 2017; Potze et al. 2014; X. Wang et al. 2017; Xu et al. 2017; H. Zhang et al. 2024). UA, by contrast, selectively triggered LMP without inducing persistent dysfunction, ultimately preserving long‐term proliferation. It selectively released cathepsins into the cytosol, triggering apoptotic pathways while preserving lysosomal recovery. UA's ability to preserve keratinocyte growth while selectively inducing loss of lysosome integrity and death in cancer cells (Conway et al. 2021; Fogde et al. 2022; Ou et al. 2014; Shin et al. 2012) positions it as a promising candidate for targeted cancer therapies. Furthermore, UA promoted efficient mitophagy, with targeted LC3‐II recruitment to COXIV‐labeled mitochondria, consistent with PRKN‐independent pathways such as AKT/mTOR/PINK1 (Castrejón‐Jiménez et al. 2019). These differential pathways suggest that membrane destabilization underlies the distinct biological outcomes of each compound.

Notably, BA can impair proton retention, leading to autophagy impairment. Despite this, CTSB and CTSL activity remained elevated over time, implying that BA does not inhibit these hydrolases. One mechanistic hypothesis center on BA's interaction with bis(monoacylglycerol)phosphate (BMP), a negatively charged lipid that recruits enzymes like acid sphingomyelinase (ASM), which has positively charged regions at acidic lysosomal pH due to its isoelectric point (pI) of about 6.8 (Kölzer et al. 2004). Similar to certain cationic amphiphilic drugs, BA may inhibit ASM, which requires BMP binding at acidic pH, without affecting CTSB or CTSL, whose isoelectric points are lower, allowing BMP‐independent activity (Hurwitz et al. 1994; Towatari et al. 1979). This hypothesis warrants further investigation and could enhance our understanding of BA's role in lysosomal modulation.

UA can disrupt lysosomal integrity in breast malignant cells by elevating lysosomal pH hours before plasma membrane damage, triggering selective lysophagy to remove damaged lysosomes (Fogde et al. 2022). When lysosomal repair fails, the activity of ASM and other hydrolases is compromised, leading to lipid accumulation, LMP, and the release of cathepsins (CTSD and CTSB) into the cytosol. Additionally, it disrupts lysosomal function, neutralizes lysosomal pH, and impairs the fusion of autophagosomes with lysosomes, resulting in the accumulation of immature autophagic structures. This cascade culminates in disrupted autophagy, which precedes apoptosis and contributes to apoptosis‐independent pathways (Fogde et al. 2022).

In malignant cell lines, BA consistently displayed more potent cytotoxicity than UA, reducing viability below 45% in nearly all tested models, supporting previous studies (Gu et al. 2017; Leal et al. 2012; Pal et al. 2015; Santos et al. 2010; Wróblewska‐Łuczka et al. 2022, 2023). However, lysosomal accumulation induced by BA varied by tumor type and did not always correlate with its cytotoxic profile. For example, SKMEL‐28 and A549 both exhibited substantial reductions in viability, yet differed in the intensity of lysosomal stress, suggesting that additional pathways, such as mitochondrial or autophagic dysfunction, are involved in BA‐induced cell death.

The UA's ability to trigger transient lysosomal damage, while allowing autophagy‐dependent repair, highlights the cytoprotective nature of autophagy in nonmalignant cells. However, this same protective mechanism may limit its cytotoxicity against certain tumors, like PC3, consistent with previous findings (Meng et al. 2015; Park et al. 2013). Cotreatment with CQ further illuminated these divergent responses. In SKMEL‐28 and HaCaT, UA plus CQ induced more substantial lysosomal accumulation than BA alone, underscoring how inhibition of lysosomal clearance can alter cell susceptibility. Additionally, CQ significantly potentiated UA‐induced cytotoxicity in A549, PC3, and SKMEL‐28 likely by disrupting protective autophagy pathways, which corroborates previous findings using carcinoma and melanomas cells (Junco et al. 2015; Lin et al. 2019; M. Wang et al. 2020). Notably, MCF7 cells exhibited a unique behavior: BA‐induced acidic vacuole accumulation remained unchanged by CQ, suggesting that lysosomal impairment was already maximal or CQ‐resistant in this context. Intriguingly, while CQ sensitized cancer cells to UA or BA, it also mitigated BA's prolonged toxicity in keratinocytes—possibly by reducing sustained lysosomal stress—reinforcing its therapeutic potential in combination protocols.

Biophysical data substantiated the functional findings, showing that BA disrupts both synthetic and mitochondria‐like membranes (POPC:CL), consistent with its disruptive cellular profile. CL, a key component of the inner mitochondrial membrane (Zorova et al. 2018), is essential for mitochondrial structure, respiratory chain activity, and mitophagy signaling (Dudek 2017). Externalized CL on the outer membrane interacts with mitophagy regulators, such as Beclin 1 and LC3, recruiting autophagic machinery (Chu et al. 2013; Huang et al. 2012). While both triterpenoids interact with mitochondria‐mimicking membranes, only BA disrupts membrane permeability.

BA, with a greater number of rotatable bonds, exhibits stronger interactions with mimetic membranes—altering membrane fluidity and increasing permeability—thereby amplifying its biological activity (Y. Chen et al. 2011; Gao et al. 2014; Martins et al. 2015, 2017; Wishart et al. 2018). In contrast, UA has fewer rotatable bonds but features a rigid hydrophobic backbone and polar functional groups, allowing it to embed in a parallel orientation within POPC‐rich bilayers. This orientation causes significant distortion of the lipid matrix, without necessarily compromising membrane integrity (Fajardo‐Sánchez et al. 2017; Wishart et al. 2018). In human erythrocytes, BA induced echinocytosis—indicative of strong membrane perturbation—while UA primarily caused cell shrinkage without topological changes (Gao et al. 2014; Jilani et al. 2011).

Together, our findings support the notion that cytotoxic selectivity is guided mainly by membrane composition and compound specificity. These results highlight two complementary therapeutic profiles: BA as a potent inducer of AACD, especially effective in aggressive tumor models, and UA as a selective modulator of autophagy, whose cytotoxicity can be enhanced through lysosomal cotargeting. Notably, the variability in lysosomal stress and recovery across cell types underscores the need for context‐specific strategies when employing triterpenoid‐based therapies. Further investigation into cell line‐specific vulnerabilities and optimal combination regimens may reveal the full potential of these compounds for targeted cancer treatment.

Collectively, our findings underscored that cytotoxic selectivity is shaped not only by compound structure but also by the lipid composition of cellular membranes. This study delineated two distinct therapeutic archetypes: BA, a robust inducer of AACD, particularly effective against aggressive tumor phenotypes; and UA, a more selective autophagy modulator whose cytotoxic potential can be amplified through lysosomal cotargeting. The observed variability in lysosomal stress responses and recovery across cell types reinforces the need for tailored therapeutic approaches. Deeper exploration of lineage‐specific vulnerabilities and strategic combinations may unlock the full therapeutic potential of these triterpenoids in precision oncology.

## 5 Conclusion

This study reveals how the distinct interactions of UA and BA with mitochondrial and lysosomal membranes dictate their differential impact on autophagy and cell fate in nonmalignant and malignant cells. UA's ability to preserve keratinocyte growth capacity, supported by cytoprotective autophagy, highlights its potential as a promising candidate for targeted cancer therapy. While BA's more indiscriminate cytotoxicity may limit its use, its potent activity of inducing AACD could still be utilized to treat aggressive cancers through a combined protocol with CQ, in which BA's prolonged adverse effects on nonmalignant cells may be mitigated. These insights provide a framework for rationally designing novel triterpenoid‐based autophagy modulators and combination strategies to enhance cancer selectivity and overcome treatment resistance. With further optimizations to improve tumor targeting and efficacy while minimizing toxicity to normal tissues, these triterpenoid‐based approaches hold promise for improving outcomes in cancer patients. The study's insights into how the biophysical interactions of triterpenoids with membranes differentially impact mitophagy and autophagy provide a basis for rationally designing new agents that modulate autophagy.

## Author Contributions

**Waleska Kerllen Martins:** conceptualization, data collection, investigation, formal analysis, interpretation of results, funding acquisition, methodology, project's design, supervision, validation, writing – review and editing. **Tayana Mazin Tsubone:** investigation, data collection, formal analysis, validation. **Chimara Emilia Nascimento Sanches:** revision and bibliographic searching. **Cleidiane de Sousa Rocha:** investigation. **Ricardo Scarparo Navarro:** writing – review and editing. **Beatriz Simonsen Stolf:** investigation, writing – editing. **Susana Nogueira Diniz:** investigation, writing – editing. **Mauricio S. Baptista:** assisted research, writing – review and editing. **Rosangela Itri:** assisted research, writing – review and editing.

## Conflicts of Interest

The authors declare no conflicts of interest.

## Supporting information


**Supporting Table 1:** The physicochemical properties of triterpenoids were obtained from DrugBank (https://go.drugbank.com/).

**Supporting Table 2:** The complete viability and lysosomal accumulation data for all seven cell lines are available in Supplementary Table 2. We treated human malignant cell lines (A549, HeLa, MES‐SA, PC3, SKMEL‐25, and SKMEL‐28) with 20 µM triterpenoids or 0.25% (v/v) DMSO for 24 h. After a 48‐h recovery period following the 24‐h treatment, we represented the cell viability of triterpenoid‐treated cells as a percentage of the DMSO control, measured by the MTT assay. Lysosomal accumulation in treated cells was represented as a fold change compared to the DMSO control. We obtained results from at least three independent experiments (*n* = 3) and expressed them as mean values ± standard error. Significance levels are indicated as *p < 0.05, **p < 0.01, ***p < 0.001.

## Data Availability

The data supporting this study's findings are available from the corresponding author upon reasonable request.

## References

[cbin70073-bib-0001] Angelova, M. I. , and D. S. Dimitrov . 1986. “Liposome Electroformation.” Faraday Discussions of the Chemical Society 81: 303–311.

[cbin70073-bib-0003] Castrejón‐Jiménez, N. S. , K. Leyva‐Paredes , S. L. Baltierra‐Uribe , et al. 2019. “Ursolic and Oleanolic Acids Induce Mitophagy in A549 Human Lung Cancer Cells.” Molecules 24: 3444. 10.3390/molecules24193444. 31547522 PMC6803966

[cbin70073-bib-0004] Chen, Y. , R. Sun , and B. Wang . 2011. “Monolayer Behavior of Binary Systems of Betulinic Acid and Cardiolipin: Thermodynamic Analyses of Langmuir Monolayers and AFM Study of Langmuir–Blodgett Monolayers.” Journal of Colloid and Interface Science 353, no. 1: 294–300. 10.1016/j.jcis.2010.09.019. 20888569

[cbin70073-bib-0005] Chen, X. , X. Yuan , Z. Zhang , et al. 2020. “Betulinic Acid Inhibits Cell Proliferation and Migration in Gastric Cancer by Targeting the NF‐κB/VASP Pathway.” European Journal of Pharmacology 889: 173493. 10.1016/j.ejphar.2020.173493. 32860808

[cbin70073-bib-0006] Chen, F. , Z. Zhong , H. Y. Tan , et al. 2020. “Suppression of lncRNA MALAT1 by Betulinic Acid Inhibits Hepatocellular Carcinoma Progression by Targeting IAPs via miR‐22‐3p.” Clinical and Translational Medicine 10: e190. 10.1002/ctm2.190. 33135336 PMC7586994

[cbin70073-bib-0007] Chu, C. T. , J. Ji , R. K. Dagda , et al. 2013. “Cardiolipin Externalization to the Outer Mitochondrial Membrane Acts as an Elimination Signal for Mitophagy in Neuronal Cells.” Nature Cell Biology 15: 1197–1205. 10.1038/ncb2837. 24036476 PMC3806088

[cbin70073-bib-0008] Conway, G. E. , D. Zizyte , J. R. M. Mondala , et al. 2021. “Ursolic Acid Inhibits Collective Cell Migration and Promotes JNK‐Dependent Lysosomal Associated Cell Death in Glioblastoma Multiforme Cells.” Pharmaceuticals 14, no. 2: 91. 10.3390/ph14020091. 33530486 PMC7911358

[cbin70073-bib-0009] Coricovac, D. , C. A. Dehelean , I. Pinzaru , et al. 2021. “Assessment of Betulinic Acid Cytotoxicity and Mitochondrial Metabolism Impairment in a Human Melanoma Cell Line.” International Journal of Molecular Sciences 22, no. 9: 4870. 10.3390/ijms22094870. 34064489 PMC8125295

[cbin70073-bib-0010] Dudek, J. 2017. “Role of Cardiolipin in Mitochondrial Signaling Pathways.” Frontiers in Cell and Developmental Biology 5: 90. 10.3389/fcell.2017.00090. 29034233 PMC5626828

[cbin70073-bib-0011] Duval, R. , P. O. Harmand , C. Jayat‐Vignoles , et al. 2008. “Differential Involvement of Mitochondria During Ursolic Acid‐Induced Apoptotic Process in HaCaT and M4Beu Cells.” Oncology Reports 19, no. 1: 145–149. 18097588

[cbin70073-bib-0012] El‐Baba, C. , A. Baassiri , G. Kiriako , et al. 2021. “Terpenoids' Anti‐Cancer Effects: Focus on Autophagy.” Apoptosis 26: 491–511. 10.1007/s10495-021-01684-y. 34269920

[cbin70073-bib-0013] Fajardo‐Sánchez, E. , V. Galiano , and J. Villalaín . 2017. “Location of the Bioactive Pentacyclic Triterpene Ursolic Acid in the Membrane. A Molecular Dynamics Study.” Journal of Biomolecular Structure and Dynamics 35, no. 12: 2688–2700. 10.1080/07391102.2016.1229219. 27569018

[cbin70073-bib-0014] Ferlay, J. , M. Ervik , F. Lam , et al. 2024. Global Cancer Observatory: Cancer Today. International Agency for Research on Cancer. https://gco.iarc.who.int/today.

[cbin70073-bib-0015] Fogde, D. L. , C. P. R. Xavier , K. Balnytė , et al. 2022. “Ursolic Acid Impairs Cellular Lipid Homeostasis and Lysosomal Membrane Integrity in Breast Carcinoma Cells.” Cells 11, no. 24: 4079. 10.3390/cells11244079. 36552844 PMC9776894

[cbin70073-bib-0017] Galluzzi, L. , N. Zamzami , T. de La Motte Rouge , C. Lemaire , C. Brenner , and G. Kroemer . 2007. “Methods for the Assessment of Mitochondrial Membrane Permeabilization in Apoptosis.” Apoptosis 12, no. 5: 803–813. 10.1007/s10495-007-0720-1. 17294081

[cbin70073-bib-0018] Gao, M. , P. M. Lau , and S. K. Kong . 2014. “Mitochondrial Toxin Betulinic Acid Induces In Vitro Eryptosis in Human Red Blood Cells Through Membrane Permeabilization.” Archives of Toxicology 88, no. 3: 755–768. 10.1007/s00204-013-1162-x. 24241250

[cbin70073-bib-0019] Garza‐Lombó, C. , A. Pappa , M. I. Panayiotidis , and R. Franco . 2020. “Redox Homeostasis, Oxidative Stress and Mitophagy.” Mitochondrion 51: 105–117. 10.1016/j.mito.2020.01.002. 31972372 PMC7062406

[cbin70073-bib-0022] Gu, W. , X. Y. Jin , D. D. Li , S. F. Wang , X. B. Tao , and H. Chen . 2017. “Design, Synthesis and In Vitro Anticancer Activity of Novel Quinoline and Oxadiazole Derivatives of Ursolic Acid.” Bioorganic & Medicinal Chemistry Letters 27, no. 17: 4128–4132. 10.1016/j.bmcl.2017.07.033. 28733083

[cbin70073-bib-0023] Hanahan, D. 2022. “Hallmarks of Cancer: New Dimensions.” Cancer Discovery 12, no. 1: 31–46. 10.1158/2159-8290.CD-21-1059. 35022204

[cbin70073-bib-0024] Huang, W. , W. Choi , W. Hu , et al. 2012. “Crystal Structure and Biochemical Analyses Reveal Beclin 1 as a Novel Membrane Binding Protein.” Cell Research 22, no. 3: 473–489. 10.1038/cr.2012.24. 22310240 PMC3292424

[cbin70073-bib-0025] Hurwitz, R. , K. Ferlinz , and K. Sandhoff . 1994. “The Tricyclic Antidepressant Desipramine Causes Proteolytic Degradation of Lysosomal Sphingomyelinase in Human Fibroblasts.” Biological Chemistry Hoppe‐Seyler 375, no. 7: 447–450. 10.1515/bchm3.1994.375.7.447. 7945993

[cbin70073-bib-0026] Iacobucci, D. , S. Román , S. Moon , and D. Rouziès . 2025. “A Tutorial on What to Do With Skewness, Kurtosis, and Outliers: New Insights to Help Scholars Conduct and Defend Their Research.” Psychology & Marketing 42: 1398–1414. 10.1002/mar.22187.

[cbin70073-bib-0027] Jilani, K. , M. Abed , C. Zelenak , E. Lang , S. M. Qadri , and F. Lang . 2011. “Triggering of Erythrocyte Cell Membrane Scrambling by Ursolic Acid.” Journal of Natural Products 74, no. 10: 2181–2186. 10.1021/np2005133. 21923134

[cbin70073-bib-0028] Junco, J. J. , A. Mancha‐Ramirez , G. Malik , et al. 2015. “Ursolic Acid and Resveratrol Synergize With Chloroquine to Reduce Melanoma Cell Viability.” Melanoma Research 25, no. 2: 103–112. 10.1097/CMR.0000000000000137. 25647735

[cbin70073-bib-0029] Klionsky, D. J. , A. K. Abdel‐Aziz , S. Abdelfatah , et al. 2021. “Guidelines for the Use and Interpretation of Assays for Monitoring Autophagy (4th Edition)^1^ .” Autophagy 17, no. 1: 1–382. 10.1080/15548627.2020.1797280. 33634751 PMC7996087

[cbin70073-bib-0030] Kölzer, M. , N. Werth , and K. Sandhoff . 2004. “Interactions of Acid Sphingomyelinase and Lipid Bilayers in the Presence of the Tricyclic Antidepressant Desipramine.” FEBS Letters 559, no. 1–3: 96–98. 10.1016/S0014-5793(04)00033-X. 14960314

[cbin70073-bib-0032] Leal, A. S. , R. Wang , J. A. R. Salvador , and Y. Jing . 2012. “Synthesis of Novel Ursolic Acid Heterocyclic Derivatives With Improved Abilities of Antiproliferation and Induction of p53, p21waf1 and NOXA in Pancreatic Cancer Cells.” Bioorganic & Medicinal Chemistry 20, no. 19: 5774–5786. 10.1016/j.bmc.2012.08.010. 22959527

[cbin70073-bib-0033] Lee, N. R. , R. Y. Meng , S. Y. Rah , et al. 2020. “Reactive Oxygen Species‐Mediated Autophagy by Ursolic Acid Inhibits Growth and Metastasis of Esophageal Cancer Cells.” International Journal of Molecular Sciences 21, no. 24: 9409. 10.3390/ijms21249409. 33321911 PMC7764507

[cbin70073-bib-0034] Lena, A. , M. Rechichi , A. Salvetti , et al. 2009. “Drugs Targeting the Mitochondrial Pore Act as Citotoxic and Cytostatic Agents in Temozolomide‐Resistant Glioma Cells.” Journal of Translational Medicine 7: 13. 10.1186/1479-5876-7-13. 19196452 PMC2661321

[cbin70073-bib-0035] Leng, S. , Y. Hao , D. Du , et al. 2013. “Ursolic Acid Promotes Cancer Cell Death by Inducing Atg5‐dependent Autophagy.” International Journal of Cancer 133, no. 12: 2781–2790. 10.1002/ijc.28301. 23737395

[cbin70073-bib-0036] Lewinska, A. , J. Adamczyk‐Grochala , E. Kwasniewicz , A. Deregowska , and M. Wnuk . 2017. “Ursolic Acid‐Mediated Changes in Glycolytic Pathway Promote Cytotoxic Autophagy and Apoptosis in Phenotypically Different Breast Cancer Cells.” Apoptosis 22, no. 6: 800–815. 10.1007/s10495-017-1353-7. 28213701 PMC5401707

[cbin70073-bib-0037] Lin, C. W. , H. K. Chin , S. L. Lee , et al. 2019. “Ursolic Acid Induces Apoptosis and Autophagy in Oral Cancer Cells.” Environmental Toxicology 34, no. 9: 983–991. 10.1002/tox.22769. 31062913

[cbin70073-bib-0038] Liu, W. , S. Li , Z. Qu , et al. 2019. “Betulinic Acid Induces Autophagy‐Mediated Apoptosis Through Suppression of the PI3K/AKT/mTOR Signaling Pathway and Inhibits Hepatocellular Carcinoma.” American Journal of Translational Research 11, no. 11: 6952–6964. 31814899 PMC6895530

[cbin70073-bib-0039] Livesey, J. H. 2007. “Kurtosis Provides a Good Omnibus Test for Outliers in Small Samples.” Clinical Biochemistry 40, no. 13–14: 1032–1036. 10.1016/j.clinbiochem.2007.04.003. 17499683

[cbin70073-bib-0040] Martins, W. K. , R. Belotto , M. N. Silva , et al. 2021. “Autophagy Regulation and Photodynamic Therapy: Insights to Improve Outcomes of Cancer Treatment.” Frontiers in Oncology 10: 610472. 10.3389/fonc.2020.610472. 33552982 PMC7855851

[cbin70073-bib-0041] Martins, W. K. , É. T. Costa , M. C. Cruz , et al. 2015. “Parallel Damage in Mitochondrial and Lysosomal Compartments Promotes Efficient Cell Death With Autophagy: The Case of the Pentacyclic Triterpenoids.” Scientific Reports 5: 12425. 10.1038/srep12425. 26213355 PMC4515638

[cbin70073-bib-0042] Martins, W. K. , C. M. Fader , E. Morselli , and D. Grasso . 2021. “Editorial: New Roles of Autophagy Pathways in Cancer.” Frontiers in Oncology 11: 726989. 10.3389/fonc.2021.726989. 34307182 PMC8294185

[cbin70073-bib-0043] Martins, W. K. , A. B. Gomide , É. T. Costa , et al. 2017. “Membrane Damage by Betulinic Acid Provides Insights Into Cellular Aging.” Biochimica et Biophysica Acta (BBA) ‐ General Subjects 1861: 3129–3143. 10.1016/j.bbagen.2016.10.018. 27773704

[cbin70073-bib-0044] Martins, W. K. , N. F. Santos , C. S. Rocha , et al. 2019. “Parallel Damage in Mitochondria and Lysosomes Is an Efficient Way to Photoinduce Cell Death.” Autophagy 15, no. 2: 259–279. 10.1080/15548627.2018.1515609. 30176156 PMC6333451

[cbin70073-bib-0045] Martins, W. K. , D. Severino , C. Souza , B. S. Stolf , and M. S. Baptista . 2013. “Rapid Screening of Potential Autophagic Inductor Agents Using Mammalian Cell Lines.” Biotechnology Journal 8, no. 6: 730–737. 10.1002/biot.201200306. 23420785

[cbin70073-bib-0046] Martins, W. K. , M. N. Silva , K. Pandey , et al. 2021. “Autophagy‐Targeted Therapy to Modulate Age‐Related Diseases: Success, Pitfalls, and New Directions.” Current Research in Pharmacology and Drug Discovery 2: 100033. 10.1016/j.crphar.2021.100033. 34909664 PMC8663935

[cbin70073-bib-0047] Meng, Y. , Z. M. Lin , N. Ge , D. L. Zhang , J. Huang , and F. Kong . 2015. “Ursolic Acid Induces Apoptosis of Prostate Cancer Cells via the PI3K/Akt/mTOR Pathway.” American Journal of Chinese Medicine 43, no. 7: 1471–1486. 10.1142/S0192415X15500834. 26503559

[cbin70073-bib-0048] Mizushima, N. , and B. Levine . 2020. “Autophagy in Human Diseases.” New England Journal of Medicine 383: 1564–1576. 10.1056/NEJMra2022774. 33053285

[cbin70073-bib-0049] Narendra, D. , A. Tanaka , D. F. Suen , and R. J. Youle . 2008. “Parkin Is Recruited Selectively to Impaired Mitochondria and Promotes Their Autophagy.” Journal of Cell Biology 183, no. 5: 795–803. 10.1083/jcb.200809125. 19029340 PMC2592826

[cbin70073-bib-0050] National Center for Biotechnology Information . 2024a. “PubChem Compound Summary for CID 64945, Ursolic Acid.” https://pubchem.ncbi.nlm.nih.gov/compound/Ursolic-Acid.

[cbin70073-bib-0051] National Center for Biotechnology Information . 2024b. “PubChem Compound Summary for CID 64971, Betulinic Acid.” https://pubchem.ncbi.nlm.nih.gov/compound/Betulinic-Acid.

[cbin70073-bib-0052] Navanesan, S. , N. Abdul Wahab , S. Manickam , Y. L. Cheow , and K. S. Sim . 2017. “Intrinsic Capabilities of *Leptospermum javanicum* in Inducing Apoptosis and Suppressing the Metastatic Potential of Human Lung Carcinoma Cells.” Chemico‐Biological Interactions 273: 37–47. 10.1016/j.cbi.2017.05.022. 28578903

[cbin70073-bib-0053] Ou, X. , M. Liu , H. Luo , L. Q. Dong , and F. Liu . 2014. “Ursolic Acid Inhibits Leucine‐Stimulated mTORC1 Signaling by Suppressing mTOR Localization to Lysosome.” PLoS One 9: e95393. 10.1371/journal.pone.0095393. 24740400 PMC3989317

[cbin70073-bib-0054] Pal, A. , A. Ganguly , S. Chowdhuri , et al. 2015. “Bis‐Arylidene Oxindole‐Betulinic Acid Conjugate: A Fluorescent Cancer Cell Detector With Potent Anticancer Activity.” ACS Medicinal Chemistry Letters 6, no. 5: 612–616. 10.1021/acsmedchemlett.5b00095. 26005543 PMC4434463

[cbin70073-bib-0055] Park, J. H. , H. Y. Kwon , E. J. Sohn , et al. 2013. “Inhibition of Wnt/β‐Catenin Signaling Mediates Ursolic Acid‐Induced Apoptosis in PC‐3 Prostate Cancer Cells.” Pharmacological Reports 65, no. 5: 1366–1374. 10.1016/S1734-1140(13)71495-6. 24399733

[cbin70073-bib-0081] Pendergrass, W. , N. Wolf , and M. Poot . 2004. “Efficacy of MitoTracker Green™ and CMXrosamine to Measure Changes in Mitochondrial Membrane Potentials in Living Cells and Tissues.” Cytometry Part A 61A, no. 2: 162–169. 10.1002/cyto.a.20033. 15382028

[cbin70073-bib-0056] Potze, L. , F. B. Mullauer , S. Colak , J. H. Kessler , and J. P. Medema . 2014. “Betulinic Acid‐Induced Mitochondria‐Dependent Cell Death Is Counterbalanced by an Autophagic Salvage Response.” Cell Death & Disease 5, no. 4: e1169. 10.1038/cddis.2014.139. 24722294 PMC5424116

[cbin70073-bib-0057] Qin, Y. , M. Ashrafizadeh , V. Mongiardini , et al. 2023. “Autophagy and Cancer Drug Resistance in Dialogue: Pre‐Clinical and Clinical Evidence.” Cancer Letters 570: 216307. 10.1016/j.canlet.2023.216307. 37451426

[cbin70073-bib-0082] Rodrigues, D. , A. C. Viotto , R. Checchia , et al. 2016. “Mechanism of Aloe Vera Extract Protection Against UVA: Shelter of Lysosomal Membrane Avoids Photodamage.” Photochemical & Photobiological Sciences 15, no. 3: 334–350. 10.1039/c5pp00409h. 26815913

[cbin70073-bib-0058] Santos, R. C. , J. A. R. Salvador , S. Marín , M. Cascante , J. N. Moreira , and T. C. P. Dinis . 2010. “Synthesis and Structure–Activity Relationship Study of Novel Cytotoxic Carbamate and *N*‐Acylheterocyclic Bearing Derivatives of Betulin and Betulinic Acid.” Bioorganic & Medicinal Chemistry 18, no. 12: 4385–4396. 10.1016/j.bmc.2010.04.085. 20494586

[cbin70073-bib-0059] Shen, S. , Y. Zhang , R. Zhang , X. Tu , and X. Gong . 2014. “Ursolic Acid Induces Autophagy in U87MG Cells via ROS‐Dependent Endoplasmic Reticulum Stress.” Chemico‐Biological Interactions 218: 28–41. 10.1016/j.cbi.2014.04.017. 24802810

[cbin70073-bib-0060] Shin, S. W. , S. Y. Kim , and J. W. Park . 2012. “Autophagy Inhibition Enhances Ursolic Acid‐Induced Apoptosis in PC3 Cells.” Biochimica et Biophysica Acta (BBA) ‐ Molecular Cell Research 1823, no. 2: 451–457. 10.1016/j.bbamcr.2011.10.014. 22178132

[cbin70073-bib-0061] Sun, C. Y. , D. Cao , Q. N. Ren , et al. 2021. “Combination Treatment With Inhibitors of ERK and Autophagy Enhances Antitumor Activity of Betulinic Acid in Non‐Small‐Cell Lung Cancer In Vivo and In Vitro.” Frontiers in Pharmacology 12: 684243. 10.3389/fphar.2021.684243. 34267658 PMC8275840

[cbin70073-bib-0062] Tonolli, P. N. , W. K. Martins , H. C. Junqueira , et al. 2020. “Lipofuscin in Keratinocytes: Production, Properties, and Consequences of the Photosensitization With Visible Light.” Free Radical Biology and Medicine 160: 277–292. 10.1016/j.freeradbiomed.2020.08.002. 32810634

[cbin70073-bib-0063] Towatari, T. , Y. Kawabata , and N. Katunuma . 1979. “Crystallization and Properties of Cathepsin B From Rat Liver.” European Journal of Biochemistry 102, no. 1: 279–289. http://www.ncbi.nlm.nih.gov/pubmed/42540. 42540 10.1111/j.1432-1033.1979.tb06290.x

[cbin70073-bib-0065] Tsubone, T. M. , C. S. Rocha , P. N. Tonolli , et al. 2020. “In Vitro Autophagy Modulation With Chloroquine: Some Lessons to Learn.” Advances in Biochemistry and Biotechnology 5: 1098. 10.29011/2574-7258.001098.

[cbin70073-bib-0066] Wang, X. , X. Lu , R. Zhu , et al. 2017. “Betulinic Acid Induces Apoptosis in Differentiated PC12 Cells via ROS‐Mediated Mitochondrial Pathway.” Neurochemical Research 42, no. 4: 1130–1140. 10.1007/s11064-016-2147-y. 28124213

[cbin70073-bib-0067] Wang, S. , K. Wang , C. Zhang , et al. 2017. “Overaccumulation of p53‐Mediated Autophagy Protects Against Betulinic Acid‐Induced Apoptotic Cell Death in Colorectal Cancer Cells.” Cell Death & Disease 8, no. 10: e3087. 10.1038/cddis.2017.485. 28981110 PMC5682653

[cbin70073-bib-0068] Wang, M. , H. Yu , R. Wu , et al. 2020. “Autophagy Inhibition Enhances the Inhibitory Effects of Ursolic Acid on Lung Cancer Cells.” International Journal of Molecular Medicine 46, no. 5: 1816–1826. 10.3892/ijmm.2020.4714. 32901853 PMC7521584

[cbin70073-bib-0069] Wang, Z. , P. Zhang , H. Jiang , B. Sun , H. Luo , and A. Jia . 2022. “Ursolic Acid Enhances the Sensitivity of MCF‐7 and MDA‐MB‐231 Cells to Epirubicin by Modulating the Autophagy Pathway.” Molecules 27, no. 11: 3399. 10.3390/molecules27113399. 35684339 PMC9182048

[cbin70073-bib-0070] Wishart, D. S. , Y. D. Feunang , A. C. Guo , et al. 2018. “DrugBank 5.0: A Major Update to the DrugBank Database for 2018.” Nucleic Acids Research 46, no. D1: D1074–D1082. 10.1093/nar/gkx1037. 29126136 PMC5753335

[cbin70073-bib-0071] Wróblewska‐Łuczka, P. , J. Cabaj , W. Bąk , et al. 2022. “Additive Interactions Between Betulinic Acid and Two Taxanes in In Vitro Tests Against Four Human Malignant Melanoma Cell Lines.” International Journal of Molecular Sciences 23: 9641. 10.3390/ijms23179641. 36077036 PMC9456196

[cbin70073-bib-0072] Wróblewska‐Łuczka, P. , J. Cabaj , J. Bargieł , and J. J. Łuszczki . 2023. “Anticancer Effect of Terpenes: Focus on Malignant Melanoma.” Pharmacological Reports 75, no. 5: 1115–1125. 10.1007/s43440-023-00512-1. 37515699 PMC10539410

[cbin70073-bib-0073] Xavier, C. P. R. , C. F. Lima , D. F. N. Pedro , J. M. Wilson , K. Kristiansen , and C. Pereira‐Wilson . 2013. “Ursolic Acid Induces Cell Death and Modulates Autophagy Through JNK Pathway in Apoptosis‐Resistant Colorectal Cancer Cells.” Journal of Nutritional Biochemistry 24, no. 4: 706–712. 10.1016/j.jnutbio.2012.04.004. 22841540

[cbin70073-bib-0074] Xu, T. , Q. Pang , Y. Wang , and X. Yan . 2017. “Betulinic Acid Induces Apoptosis by Regulating PI3K/Akt Signaling and Mitochondrial Pathways in Human Cervical Cancer Cells.” International Journal of Molecular Medicine 40, no. 6: 1669–1678. 10.3892/ijmm.2017.3163. 29039440 PMC5716432

[cbin70073-bib-0075] Yang, L. , Y. Chen , J. He , et al. 2012. “Betulinic Acid Inhibits Autophagic Flux and Induces Apoptosis in Human Multiple Myeloma Cells In Vitro.” Acta Pharmacologica Sinica 33, no. 12: 1542–1548. 10.1038/aps.2012.102. 23064721 PMC4001834

[cbin70073-bib-0076] Yoshimori, T. , A. Yamamoto , Y. Moriyama , M. Futai , and Y. Tashiro . 1991. “Bafilomycin A1, a Specific Inhibitor of Vacuolar‐Type H⁺‐ATPase, Inhibits Acidification and Protein Degradation in Lysosomes of Cultured Cells.” Journal of Biological Chemistry 266, no. 26: 17707–17712. 10.1016/s0021-9258(19)47429-2. 1832676

[cbin70073-bib-0078] Zhang, H. , M. Zhou , C. Ye , et al. 2024. “Betulinic Acid Inhibits the Proliferation of Human Laryngeal Carcinoma Cells Through Reactive Oxygen Species‐Mediate Mitochondrial Apoptotic Pathway.” Toxicology In Vitro 95: 105756. 10.1016/j.tiv.2023.105756. 38061603

[cbin70073-bib-0079] Zhao, C. , S. Yin , Y. Dong , et al. 2013. “Autophagy‐Dependent EIF2AK3 Activation Compromises Ursolic Acid‐Induced Apoptosis Through Upregulation of MCL1 in MCF‐7 Human Breast Cancer Cells.” Autophagy 9, no. 2: 196–207. 10.4161/auto.22805. 23182854 PMC3552883

[cbin70073-bib-0080] Zorova, L. D. , V. A. Popkov , E. Y. Plotnikov , et al. 2018. “Mitochondrial Potential.” Analytical Biochemistry 552: 50–59. 10.1016/j.ab.2017.07.009. 28711444 PMC5792320

